# A sustainable multi-zeolite synthetic framework from a single natural clay: CO_2_/H_2_O adsorption performance and life cycle assessment benefits

**DOI:** 10.1039/d5se01375e

**Published:** 2026-01-19

**Authors:** Biruktait Ayele Lemecho, Jordi Espín, Pattaraphon Rodlamul, Florian Kiefer, Wendy Lee Queen, Vivek Subramanian

**Affiliations:** a Laboratory for Advanced Fabrication Technologies (LAFT), Institute of Electrical and Micro Engineering, Ecole Polytechnique Fédérale de Lausanne (EPFL) Neuchâtel 2000 Switzerland biruktait.lemecho@epfl.ch vivek.subramanian@epfl.ch; b Laboratory for Functional Inorganic Materials (LFIM), Institut des Sciences et Ingénierie Chimiques, École Polytechnique Fédérale de Lausanne (EPFL) Rue de l'Industrie 17 1951 Sion Switzerland; c Chemical Energy Carriers and Vehicle Systems Laboratory, Empa – Swiss Federal Laboratories for Materials Science and Technology 8600 Dübendorf Switzerland

## Abstract

A novel sustainable synthesis strategy for producing a range of structurally distinct zeolites, specifically Zeolite 4A, Zeolite 13X, and Zeolite Y, is presented. This method avoids organic templates (commonly used for many high-silica zeolites such as ZSM-5, Beta, or high-silica Y) and directly produces Zeolite 4A, Zeolite 13X, and Zeolite Y from natural bentonite clay without the need for synthetic silica or alumina sources and thus offers a much more environmentally-benign production strategy than existing commercial synthetic routes. By systematically tuning alkaline fusion conditions and hydrothermal crystallization parameters, selective zeolite phase formation is achieved: lower fusion temperatures and NaOH/clay ratios favor the formation of LTA-type Zeolite 4A, while higher values promote the formation of FAU-type Zeolite 13X and Zeolite Y. The synthesized zeolites demonstrated structural characteristics and adsorption performance comparable to their commercial counterparts. Zeolite 13X exhibited the highest CO_2_ adsorption capacity, attributed to its elevated microporosity and sodium content, while Zeolite Y showed enhanced hydrothermal stability and reduced water affinity, resulting from its higher Si/Al ratio and lower cation density. Water vapor adsorption isotherms and repeated cycling tests revealed clear differences in hydrothermal stability between the synthesized zeolites. A cradle-to-gate life cycle assessment (LCA), performed for Zeolite 13X as a representative product, revealed a ∼90% reduction in global warming potential (2.48 *vs.* 24.25 kg CO_2_ eq. per kg), over 95% lower cumulative energy demand, and significantly decreased ecotoxicity and human toxicity indicators when compared to conventional chemical synthesis. Additionally, cost-oriented economic analysis showed that the clay-based synthesis route reduces the production cost of Zeolite 13X by approximately 33% compared to conventional chemical synthesis. Overall, this work provides a mechanistically informed, environmentally friendly framework for the phase-selective synthesis of industrially relevant zeolites from natural clay.

## Abstract 2

A sustainable clay-to-zeolite synthesis cuts CO_2_ emissions by 90%, enabling high-performance CO_2_/H_2_O adsorption with minimal environmental impact.

## Graphical abstract

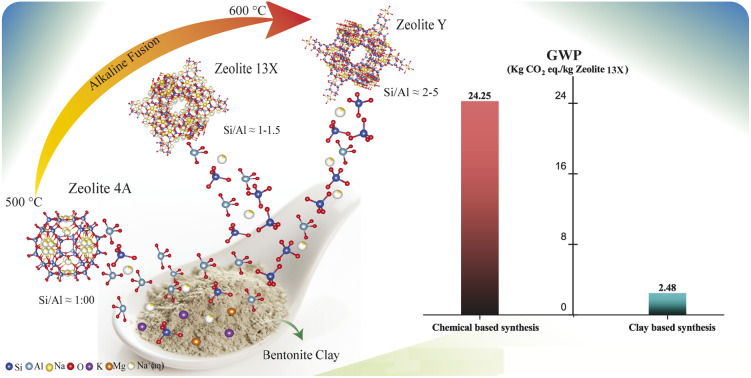

## 1 Introduction

Zeolites are crystalline, microporous aluminosilicates that are vitally important in a variety of industries including gas separation, adsorption, and catalysis, because of their exceptional ion-exchange capacity, large surface area, and tunable properties.^1,2^ Zeolite 13X, Zeolite 4A, Zeolite Y, and ZSM-5 are the most commonly used zeolite types for industrial processes such hydrocarbon conversion, water softening, and carbon dioxide (CO_2_) capture.^3–5^ Industrially, these zeolites are commonly synthesized using chemical Si and Al sources, such as sodium silicate, silica, aluminum sulfate, and sodium aluminate.^6^ However, the energy-intensive and environmentally hazardous processes required to produce these raw materials pose significant challenges to the sustainable industrial production of zeolites.^7,8^ The high carbon dioxide footprint and cost of production of these raw materials further amplifies the overall environmental and economic impact of industrial zeolite production.^9^ Given these limitations, there is a growing interest in clay-based zeolite synthesis as a viable and cost effective alternative.^10^

Natural clays are abundant, low-cost, and rich in silica and alumina, which are fundamental required components for zeolite synthesis.^11,12^ Bentonite clay, from the montmorillonite clay family, is particularly attractive due to its unique layered structure, high cation exchange capacity, and favorable chemical composition.^13–15^ Structurally, bentonite consists of stacked silica tetrahedral and alumina octahedral sheets, with a composition of approximately 71.62% SiO_2_, 15.22% Al_2_O_3_, and 13.17% water.^16,17^ Its bulk Si/Al ratio can reach values of ∼4.7, thus providing sufficient total silicon and aluminum for synthesizing a variety of zeolite types and eliminating the need for synthetic silica and alumina additives, while allowing framework composition and phase selectivity to be tuned through controlled alkaline activation and crystallization conditions.^11,18^

There is growing interest in clay-based zeolite synthesis due to its cost effectiveness and sustainability.^19^ Recent studies have demonstrated the feasibility of synthesizing zeolites directly from natural clays as alternative silicon and aluminum sources. For example, attapulgite clay has been used for the synthesis of ZSM-5 through tailored activation strategies,^20^ while kaolinite-based clays have been reported as precursors for denser zeolite frameworks such as sodalite and cancrinite under controlled synthesis conditions.^21,22^ These studies clearly demonstrate that natural clays can serve as viable raw materials for zeolite synthesis. However, despite the promise of natural clays as raw materials for production of various zeolite types, most reported studies focus on the synthesis of a single zeolite phase and often rely on additional synthetic silica or alumina to achieve the desired stoichiometry.^17,23,24^ Therefore, the current literature is missing a systematic understanding of how to control the release and availability of silicon and aluminum species directly from the clay matrix, and how these parameters govern the crystallization pathways of different zeolite structures. While alkaline activation of clays (including bentonite) is widely applied to enhance reactivity of the clay,^23,25^ there is a significant gap in studies that integrate the effect of fusion parameters (*e.g.*, alkali concentration and fusion temperature), crystallization conditions (such as time and temperature of hydrothermal treatment), and the resulting phase selectivity (the type of zeolite structure formed). Furthermore, despite frequent claims that clay-based methods are greener approaches,^14,26^ very few studies have quantitatively assessed the environmental impact of zeolite synthesis.^27^ To the best of our knowledge, studies that systematically link synthesis conditions to environmental impact through life cycle assessment (LCA) are absent from the literature. Therefore, there is a clear need for a comprehensive investigation into clay-derived zeolite synthesis mechanisms, coupled with comparative life cycle assessment (LCA) to rigorously evaluate energy consumption, greenhouse gas emissions, and the global warming potential of clay-based *versus* conventional chemical synthesis routes.

In this study, we introduced a comprehensive synthesis strategy for producing multiple zeolite types (*i.e.*, Zeolite 13X, Zeolite Y, and Zeolite 4A) from a single precursor (*i.e.*, bentonite clay) without the addition of synthetic silica or alumina, with the goal of realizing a more environmentally-benign synthetic process. A screening design of experiments (DOE) approach was used to define and study the range of synthesis parameters to ensure comprehensive coverage of conditions to yield different zeolite types. In addition to synthesis optimization, evaluating the functional performance of the synthesized zeolites under relevant operating conditions is essential. Among the various applications of zeolites, this study focuses on carbon dioxide and water vapor adsorption due to their strong industrial and environmental relevance. Carbon dioxide is a key target in carbon capture and utilization technologies,^28^ while water vapor adsorption plays a critical role in real gas separation processes, where moisture is inevitably present and can significantly influence adsorption behavior and material stability.^29,30^ Other applications such as ion-exchange-based removal of ionic pollutants, although well established for LTA-type zeolites, are beyond the scope of this study. Therefore, the resulting zeolites were structurally characterized and evaluated for their adsorption performance toward CO_2_ and H_2_O, focusing on equilibrium uptake, thermal stability, and cyclic stability under relevant operating conditions; see Methods).^31,32^ Water adsorption measurements were performed across a wide temperature range and showed distinct differences in adsorption capacity. Long-term cyclic testing under humid conditions was also demonstrated in hydrothermal testing conditions (humid atmosphere at elevated pressure and temperature (200 °C), along with complementary structural characterization performed before and after hydrothermal cyclic tests to explain the observed structural integrity trends. Furthermore, a cradle-to-gate LCA framework was developed to compare the environmental impact of the zeolites synthesized in this study against those produced using conventional chemical-based synthesis. Material and energy flows were analyzed at each stage of the synthesis, with a focus on key environmental impact categories including Global Warming Potential (GWP, kg CO_2_-eq. per kg zeolite), Cumulative Energy Demand (CED, MJ per kg zeolite) and environmental impacts related to toxicity (Comparative Toxic Units for ecosystems (CTUe) and Comparative Toxic Units for humans (CTUh)). Beyond environmental considerations, the economic viability of zeolite synthesis routes is a critical factor for large-scale deployment. Accordingly, a cost-oriented economic analysis was conducted to assess the production cost implications of clay based *versus* conventional chemical-based synthesis routes.

Overall, this work provides the first comprehensive study to enable the synthesis of multiple zeolite types from a single natural precursor (bentonite clay) with composition-driven performance evaluation spanning CO_2_ and H_2_O adsorption behavior, thermal stability, and life cycle environmental assessment, thus bridging material design, function, and sustainability in a single comprehensive study.

## 2 Materials and methods

### 2.1. Experimental

#### 2.1.1 Materials

A commercially sourced bentonite clay (Sigma-Aldrich) was used as a precursor clay for the synthesis of Zeolite 13X, Zeolite Y and Zeolite 4A. Sodium hydroxide (NaOH) pellets (Sigma-Aldrich, 995) were used for alkaline fusion treatment of the clay. For comparison, commercial Zeolite 13X, Zeolite Y and Zeolite 4A samples were used from the same supplier.

#### 2.1.2 Synthesis of Zeolite 4A, Zeolite 13X and Zeolite Y from bentonite clay

**Fig. 1:**
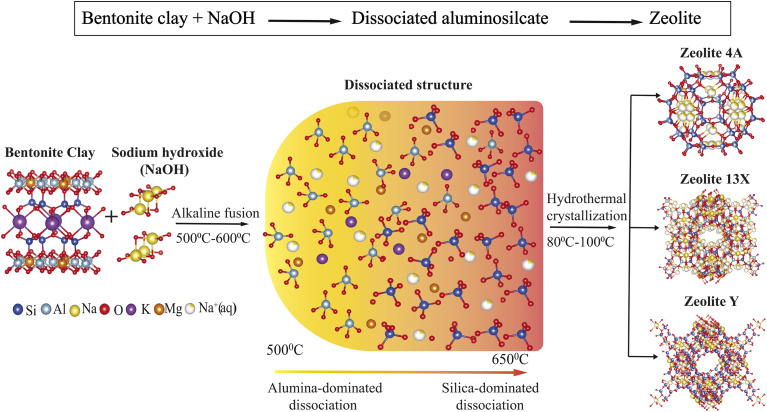
Schematic representation of the synthesis of Zeolite 4A, Zeolite 13X, and Zeolite Y from bentonite clay.

Fig. 1 illustrates the overall synthesis strategy, showing the molecular transformation pathway from the layered aluminosilicate structure of bentonite clay to the formation of different zeolite frameworks. Alkaline fusion was used to break up the clay structure. The resulting dissociated products were subjected to hydrothermal treatment to facilitate their assembly to form Zeolite 4A, Zeolite 13X, and Zeolite Y as a function of the treatment conditions. The clay served as the sole source of both silica and alumina, with no additional Si or Al precursors introduced. To systematically investigate the influence of key synthesis parameters on phase formation, a screening design of experiments (DOE) was designed and analyzed using JMP® software (by SAS Institute).^33^ A series of experiments were executed to identify the main factors controlling zeolite crystallization and to ensure efficient coverage of the design space with a minimized number of experimental runs (see SI, Table 1). The selection of experimental variables and their respective levels was guided by insights from previous studies^11,12,23^ focusing on the bentonite/NaOH ratio, alkaline fusion temperature, fusion time, stirring time, crystallization temperature, and crystallization time, as summarized in Table 1. Each factor was evaluated at two levels to systematically assess its influence on phase selectivity. The primary responses monitored during synthesis were the resulting zeolite phase type, phase purity, and degree of crystallinity.

**Table 1:** List of factors, levels and responses of the DOE

	Factors	Level		Unit	Responses
		Minimum	Maximum		
1	Bentonite/NaOH	1 : 1	1 : 1.8	wt%	Zeolite type
2	Alkaline fusion temperature	500	650	°C	
3	Alkaline fusion time	1	6	h	Phase purity
4	Stirring time	16	24	h	
5	Crystallization temperature	80	100	°C	Crystallinity
6	Crystallization time	8	24	h	

As a first step, the bentonite clay was fused using sodium hydroxide (NaOH) at varying fusion temperatures and NaOH ratios. To activate the clay structure and depolymerize its aluminosilicate layers into reactive building blocks, the clay was mixed with NaOH in different ratios (1 : 1–1 : 1.8) and fused in a muffle furnace at temperatures ranging from 500 °C to 650 °C for durations between 1 and 6 hours. The resulting fused solid was ground into a fine powder and mixed with deionized water at a controlled volume ratio, followed by vigorous stirring at room temperature for 16 to 24 hours. The obtained slurry was then transferred to a Teflon-lined autoclave and subjected to hydrothermal crystallization at temperatures between 80 °C and 100 °C for 8 to 24 hours. The final products were filtered, washed thoroughly with deionized water until reaching neutral pH, and dried overnight at 100 °C.

### 2.2. Characterization methods

#### 2.2.1 Crystallographic and topological characterization

To determine the structure of the crystallization products, X-ray Diffraction (XRD) analysis was performed. The XRD analysis of bentonite and synthesized zeolite samples were performed on a PANalytical Empyrean X-ray polycrystalline diffractometer in Bragg–Brentano geometry, equipped with a long-focus sealed Cu X-ray tube (*λ*_Kα_ = 1.5418 Å), and PIXcel 1D X-ray detector. X-ray diffraction patterns were collected between 5 and 100° (2 theta), with a step size of 0.02626°. The relative crystallinity of the selected samples was calculated by comparing their integrated peak areas to those of the corresponding commercial references (eqn (1)) according to previous reports^34^1
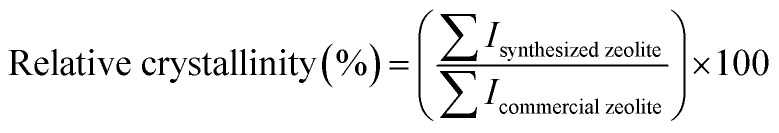
where, ∑*I*_synthesized zeolite_ represents the sum of the integrated peak areas of the main characteristic peaks of the synthesized zeolites, and ∑*I*_commercial_ corresponds to the sum of the integrated peak areas of the same peaks in the commercial reference zeolite (of the same type). Between 10 and 15 characteristic peaks unique to each zeolite structure were selected for this comparison.

Additionally, morphological analysis and chemical composition analysis of the bentonite clay and the synthesized zeolite samples were performed using scanning electron microscopy (Gemini SEM 450) along with energy dispersive X-ray spectroscopy (EDXS).

Surface area characterization is critical to understanding adsorption behavior. Nitrogen adsorption–desorption measurements were performed on Autosorb IQ-XR and Belsorp Max II (Bel Japan, Inc.) instruments. Specific surface area and pore size distribution were measured by nitrogen adsorption at 77 K (−196 °C). The nitrogen adsorption–desorption isotherms of Zeolite 4A and Zeolite 13X were measured using the Autosorb IQ-XR system, while Zeolite Y samples were measured using the Belsorp Max II instrument. Prior to the measurement, the samples were activated at 573 K (300 °C) for 3 hours under vacuum in the Autosorb IQ-XR station II and Belsorp Vac II activation stations.

#### 2.2.2 CO_2_ adsorption and isosteric heat of adsorption measurements

To evaluate carbon capture performance of the zeolites herein, CO_2_ adsorption measurements were performed. The CO_2_ adsorption measurements were performed at 298 K (25 °C) on a Belsorp max instrument (Bel Japan, Inc.). Prior to the measurement, the samples were activated at 573 K (300 °C) for 3 hours under vacuum, as above. To determine the isosteric enthalpies of adsorption, CO_2_ adsorption isotherms were collected at 313, 333, and 353 K and fitted to a dual-site Langmuir model following eqn (2).2
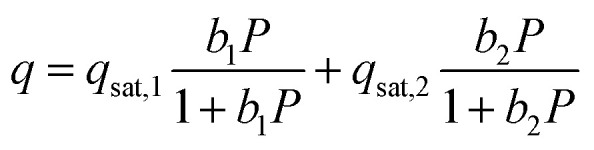
where *q* is the adsorbed amount in mmol g^−1^, *q*_sat,1_ is the adsorption capacity for site 1, *b*_1_ is the Langmuir parameter for site 1 (*q*_sat,2_ and *b*_2_ are the equivalent for site 2) and *P* is the pressure in Pa. Next, the Clausius–Clapeyron equation (eqn (3)) was subsequently used to calculate the isosteric enthalpy of adsorption, *Q*_st_, for CO_2_.3
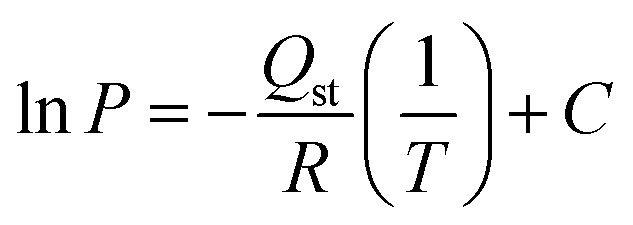


#### 2.2.3 CO_2_ adsorption cyclic stability test

For evaluating the stability of the synthesized zeolite samples as CO_2_ sorbents, thermogravimetric analysis (TGA) was performed, using a TGA Q500 instrument from TA Instruments. Specifically, to evaluate stability of CO_2_ adsorption, gas line 1 was connected to a N_2_ cylinder (balance) and gas line 2 to a CO_2_ cylinder (sample). The balance flow was set to 15 mL min^−1^ and the sample flow to 30 mL min^−1^. A 60 min pretreatment under N_2_ at 623 K (350 °C) was applied prior to the start of all the cycles. Then, pure CO_2_ gas was continuously flowed while the temperature of the furnace was switched between 298 and 623 K (25 and 350 °C), with isotherm times of 10 min at each step. Approximately 15 mg of sample was used in each test.

#### 2.2.4 Water sorption and cyclic stability tests

A custom-designed experimental setup (Fig. S3, see SI), was used to investigate water adsorption performance of the synthesized Zeolite 13X and Zeolite Y under high temperature and high pressure conditions relevant to sorption-enhanced catalysis. Breakthrough experiments were conducted to evaluate the cyclic stability and water sorption capacity of the materials. The system included a mass flow and water dosing unit composed of a Bronkhorst Flexi FLOW mass flow controller (maximum 2 NL min^−1^) for regulating nitrogen flow, and a Bronkhorst Controlled-Evaporator-Mixer (CEM) equipped with a mini CORI-FLOW M13 (maximum 120 g h^−1^) for precise water injection. Downstream of the evaporator, a Bronkhorst EL-FLOW Prestige controller (maximum 20 NL min^−1^) allowed further nitrogen dilution before the gas stream was preheated and introduced into the reactor. The adsorbent was packed into a stainless-steel double-tube reactor with an inner diameter of 10 mm, and reactor temperature was controlled using a Julabo HT 60 thermal oil system capable of reaching up to 350 °C. This system included both an electric heater and a water cooler, enabling fast and accurate temperature swings. After passing through the reactor, the gas flowed through a water-cooled condenser, followed by a Bronkhorst EL-PRESS back-pressure controller (rated up to 25 bar(a)) to maintain system pressure. A portion of the outlet gas stream ≈20 L h^−1^ was sampled through a heated line and a Hiden high-temperature/high-pressure valve, allowing direct sampling from elevated temperatures and pressures. Gas composition was analyzed using a Hiden QGA quadrupole mass spectrometer operating at ambient pressure. Prior to experiments, all samples were dried at 400 °C for 2 hours before being loaded into the reactor. A reference cycle was conducted five times before breakthrough experiments to ensure consistent baseline performance and to monitor degradation. Each cycle involved drying at 350 °C for 1 hour under a nitrogen purge of 4 NL min^−1^, followed by feeding nitrogen and water vapor at 10 bar(a) and 1 bar water partial pressure (15 g h^−1^) for 30 minutes until full breakthrough was reached, and then repeating the drying step. This cycle was repeated between isotherm measurements, which were performed under various water partial pressures and temperatures to determine the material's sorption performance.

#### 2.2.5 Life-cycle assessment (LCA)

A key goal of this work is to explicitly address the relative environmental benefits of the synthetic strategies proposed herein *vis-à-vis* conventional synthetic method. A comparative Life Cycle Assessment (LCA) was performed following the ISO 14040 (ref. 35) and ISO 14044 (ref. 36) standards to evaluate the environmental impacts of Zeolite 13X synthesis *via* two distinct routes: the clay-based method developed in this work and a chemical-based method that matches known commercial methods adapted from a well-established literature source (WO 2023/119309 A1).^37^ Given that the synthesis methodology was the same for all zeolite types and considering that Zeolite 13X is the most commonly synthesized zeolite type from clay sources, it was selected as the representative material for the LCA comparison. The LCA framework included the four standard phases: goal and scope definition, life cycle inventory (LCI), life cycle impact assessment (LCIA), and interpretation. The study was conducted using openLCA software, and life cycle inventory data was compiled from multiple sources including the Ecoinvent 3.1,^38^ USEEI,^39^ and OZLCI2019 (ref. 40) databases. Based on data availability and relevance, region-specific data were also applied from Austria, the EU, and Switzerland. The assessed environmental impact categories were: global warming potential (GWP) using the IPCC 2013 GWP 100a method,^41^ cumulative energy demand (CED),^38,42^ and human and ecotoxicity using the USEtox model.^43^ Where specific background data (*e.g.*, sodium aluminate, sodium silicate) was unavailable in standard databases, processes were constructed based on literature reported inventory data and stoichiometric calculations. The chemical-based route inventory was based on the method from the patented work used^37^ and the synthesis of precursors such as sodium silicate^44^ and sodium aluminate^45,46^ from available database proxies. Emissions and energy demands from precursor production were therefore included in the final impact calculations to ensure consistent and comprehensive comparison across the two synthesis routes.

##### 2.2.5.1. LCA goal and scope definition

**Fig. 2:**
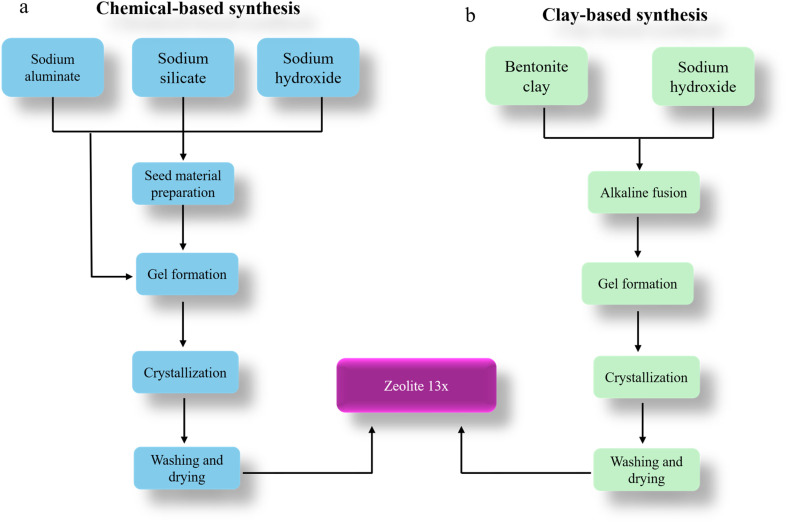
Life cycle assessment (LCA) system boundaries for the synthesis of Zeolite 13X *via* (a) a conventional chemical-based method^11^ (b) clay-based method: this work.

The primary goal of this study is to perform a comparative Life Cycle Assessment (LCA) to assess the environmental impacts associated with the synthesis of Zeolite 13X through two different approaches, namely, a natural aluminosilicate route using bentonite clay as a sustainable raw material as experimentally demonstrated in this work, and a chemical-based synthesis route, adapted from a patented literature method,^37^ which relies on conventionally used chemical precursors such as sodium silicate and sodium aluminate. This comparison aims to determine which synthesis route offers greater environmental benefits while delivering comparable material quality. The assessment was carried out using a functional unit of 1 kg of Zeolite 13X (dry weight), ensuring a consistent basis for comparing both methods. A ‘cradle-to-gate’ system boundary was the scope, which includes all relevant stages from raw material extraction and precursor production to the final zeolite synthesis and drying.^6^ The system boundary also accounts for energy consumption, transport, water use, and emissions associated with each input and process stage. The life cycle of Zeolite 13X in this study is categorized into four major stages: (1) pretreatment of raw materials, (2) gel formation and crystallization, (3) filtration and drying, and (4) product recovery. These steps were common for both chemical and clay-based synthesis routes (Fig. 2). In the chemical-based synthesis, the primary raw materials, including sodium aluminate, sodium silicate, and sodium hydroxide are first produced externally. These chemicals are then directed to a preparation step, where sodium aluminate and sodium silicate solutions are mixed to form a seed gel. This seed material is aged at 30–45 °C for 18–26 hours to cause nucleation. In parallel, a secondary gel is prepared by mixing additional sodium aluminate, sodium silicate, and NaOH with water, into which the aged seed gel is incorporated. This mixture undergoes stirring and hydrothermal crystallization at 95–100 °C for 8–12 hours. Post-crystallization, the formed Zeolite 13X is separated through filtration, washed using hot demineralized water at 90 °C to remove occluded sodium ions, and dried at 110–120 °C for 24 hours to yield Zeolite 13X powder, which is the final product.

In contrast, the clay-based synthesis begins with raw bentonite clay, which undergoes alkaline fusion with sodium hydroxide at moderately high temperatures to activate and transform the aluminosilicate structure. This fused product is then processed into a reactive gel through mixing with water and continues stirring. The crystallization step is performed under nearly similar hydrothermal conditions (80–100 °C) as the chemical route to ensure the structural development of Zeolite 13X. Afterward, filtration, washing, and drying steps are carried out, yielding Zeolite 13X with comparable crystalline quality. End-of-life disposal, product use, and equipment maintenance are excluded from the boundary as these are assumed to be similar across both systems.

##### 2.2.5.2. Life cycle inventory

The life cycle inventory phase involves the systematic quantification of energy, material inputs, and environmental releases associated with the production of Zeolite 13X *via* both chemical and clay-based synthesis routes. This was conducted by integrating the collected experimental and secondary data into a modeling framework using open LCA 2.3.1.^47^ In this study, the inventory data was modeled using multiple LCA databases, including openLCA-IW-plus-for-ei3-5,^48^ USEEI,^39^ elcd 3.2,^49^ and ecoinvent compatible datasets.^38^ A custom database was created to host specific processes including bentonite clay activation, sodium silicate production, and sodium aluminate production. These processes were individually defined and linked to background data available in the aforementioned databases. The foreground data for the clay-based synthesis was developed based on lab-scale experimental procedures performed using bentonite clay and sodium hydroxide. The process flow includes alkaline fusion, gel formation, hydrothermal crystallization, filtration, and drying. The chemical-based route, on the other hand, was modeled based on the aforementioned patent and literature-described industrial process that utilizes sodium aluminate, sodium silicate, and sodium hydroxide for seed gel preparation and final crystallization. This included precise mass ratios, reaction conditions, and process times. Background inventory data, including electricity, natural gas, steam generation, water usage, and the production and shipping of sodium hydroxide, sodium silicate, and sodium aluminate were taken from global and regional datasets available in Eco invent,^43^ elcd,^49^ and USEEI.^39^ Where exact processes were not found (*e.g.*, for sodium aluminate or silicate), proxy processes were developed with justifications provided based on the literature or environmental reports. According to the ISO 14044:2006 cut-off criterion,^36^ inputs that have less than 1 wt% of the total materials were excluded from the inventory, assuming negligible environmental impact. Similarly, particulate emissions from solid handling (*e.g.*, grinding or drying steps) were not included due to lack of quantifiable data and minimal impact on comparative results. The process structure, inputs, outputs, and emissions were validated across mass and energy balances, and the full inventory is summarized in SI (see SI Tables S2 and S3).

##### 2.2.5.3. Methods for life cycle impact analysis

The life cycle impact assessment (LCIA) provides a standardized framework for evaluating environmental impacts based on life cycle inventory (LCI) results. In this study, the methodology in the International Reference Life Cycle Data System (ILCD) Handbook^50^ was followed to quantitatively calculate selected environmental impact indicators relevant to zeolite synthesis. The assessment particularly focused on key environmental impact categories, including global warming potential (GWP, expressed as kg CO_2_ eq. per kg of Zeolite 13X), cumulative energy demand (CED, expressed as MJ per kg of Zeolite 13X), and toxicity-related impacts by including both ecotoxicity and human toxicity.^38,43,48^

Global warming potential (GWP) or carbon footprint is one of the key environmental indicators considered in life cycle assessment (LCA) studies.^6,35^ In this work, the carbon footprint was quantified as the 100-year global warming potential (GWP100), based on CO_2_-equivalent factors provided by the Intergovernmental Panel on Climate Change (IPCC).^41^ In parallel, the cumulative energy demand (CED) was evaluated to account for the total amount of energy (both renewable and non-renewable) consumed throughout the life cycle stages, including raw material production, and manufacturing.^38,51^ The energy embedded in raw materials was obtained from open-access life cycle databases, while the energy consumption during the synthesis processes was calculated based on the measured heat and electricity inputs. Heat was assumed to be supplied by natural gas combustion, and electricity consumption was modeled using the average electricity mix in Switzerland.^52^ Since these same input parameters were used for both synthetic pathways, the conclusions will translate to other geographic regions as well. Additionally, the environmental impacts related to toxicity were assessed by evaluating ecotoxicity and human toxicity categories.^38,43^ These indicators reflect the potential harm caused by emissions of toxic substances into air, water, and soil during the life cycle stages of zeolite synthesis.^6^ Ecotoxicity was quantified using Comparative Toxic Units for ecosystems (CTUe), which estimate the potential adverse effects on terrestrial and aquatic ecosystems.^43^ Human toxicity impacts were assessed using Comparative Toxic Units for humans (CTUh), which evaluate the potential risks to human health through inhalation, ingestion, or dermal exposure pathways.^43^ The toxicity characterization was conducted according to the impact assessment models embedded within the life cycle open LCA databases,^47–49^ following the ILCD methodology framework.^50^

### 2.3. Cost-oriented economic comparison

A cost-oriented economic comparison was performed by combining material and energy inventories derived from the LCA with representative unit prices, following established LCA-based economic assessment practices.^35,53,54^ Following the rationale described in the Methods section, Zeolite 13X was selected as the representative zeolite for the cost-oriented economic comparison. For each synthesis route, the total cost per kilogram of Zeolite 13X product was calculated by summing the individual contributions of material inputs and energy consumption, normalized to the functional unit of 1 kg Zeolite 13X.
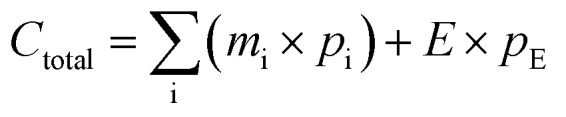
where, *m*_i_ = mass of input material i per kg zeolite (from LCA inventory), *p*_i_ = unit price of material i, *E* = electricity consumption per kg zeolite and *p*_E_ = electricity price. This approach provides a transparent, process-level comparison of relative cost drivers between synthesis routes and does not represent a full techno-economic analysis. Representative industrial market prices were used for all raw materials and utilities in both synthesis routes to reflect realistic large-scale production conditions and to ensure consistency with the LCA framework.

## 3 Results and discussion

### 3.1. Synthesis of Zeolite 13X from bentonite

To evaluate the crystalline phases of the synthesized zeolite samples, XRD analyses were performed for all synthesized samples. The results confirmed the formation of multiple zeolite types, such as Zeolite 4A, Zeolite 13X, and Zeolite Y, depending on the choice of fusion temperature, NaOH ratio, and crystallization conditions.

**Fig. 3:**
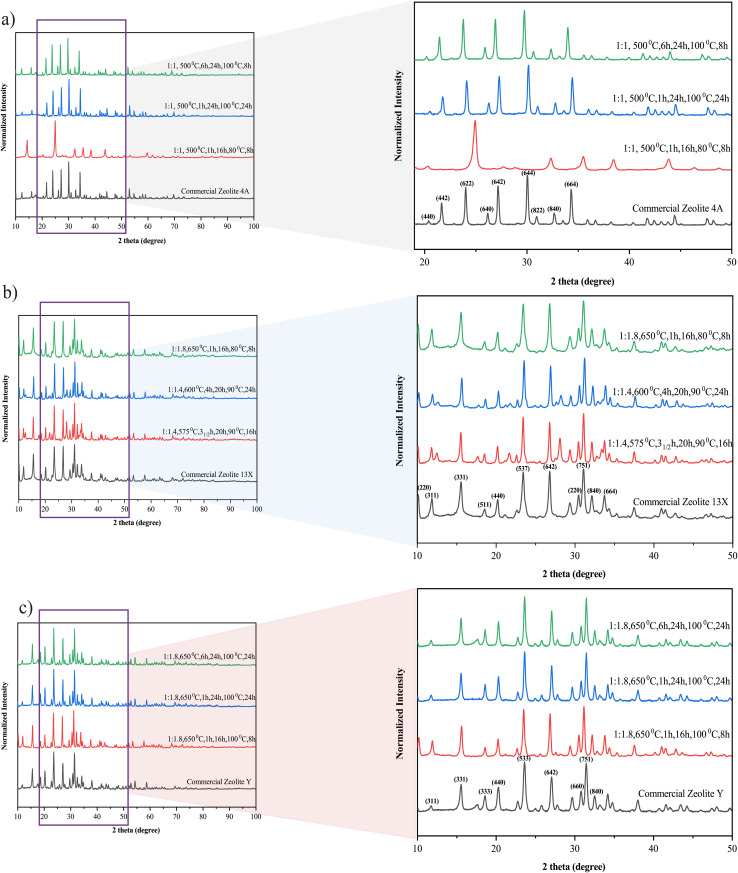
Normalized XRD patterns of synthesized zeolites compared with commercial references: (a) Zeolite 4A, (b) Zeolite 13X, (c) Zeolite Y. Sample codes represent the synthesis conditions in the format: NaOH/clay ratio, fusion temperature (°C), fusion time (h), stirring temperature (°C), stirring time (h), crystallization time (h). The boxed region is enlarged to 20–50° (4A) and 10–50° (Zeolite 13X/Y) to highlight the major peaks. An additional zoomed-in image of the high-angle region (50–100° 2*θ*) is provided in Fig. S2(b–d) (see SI) to further confirm the purity of the synthesized samples.

Representative XRD patterns of the most crystalline and phase-pure samples from each zeolite type are shown in Fig. 3(a–c), alongside their corresponding commercial counterparts for benchmarking. All patterns were normalized to their most intense peak for consistent comparison; the boxed regions are zoomed to highlight reflections. As shown in Fig. S2(a), (see SI), the XRD pattern of the bentonite clay shows a combination of montmorillonite (M) and quartz (Q) phases. A broad peak at around 6–9° and 20° (2 theta), corresponds to the *d*_(001)_ and *d*_(020)_ faces of montmorillonite respectively, indicating a layered silicate layered structure.^55^ The additional peaks at around 26° and 36°, correspond to quartz impurities, which are common in naturally occurring bentonite.^56,57^ The relatively broad and less intense peaks across the scan range confirm the semi-crystalline nature of the bentonite clay.^21^ Upon alkaline fusion, particularly at higher NaOH ratios and elevated fusion temperatures (≥650 °C), the clay structure is expected to undergo significant depolymerization, releasing reactive Si and Al species, as reported in previous reports.^25,58^ This structural breakdown facilitates the rearrangement into different zeolite frameworks during hydrothermal crystallization. At lower fusion temperatures (≈500 °C), the extent of activation remains limited, favoring the formation of low-silica phases such as Zeolite 4A,^59,60^ whereas higher temperatures promote the development of silica-rich frameworks such as Zeolite Y and Zeolite 13X.^23,61^Fig. 3(a–c), show samples exhibiting the highest phase purity within each category of zeolites and comparisons with their commercial counterparts. The sample codes and synthesis conditions for each phase are provided alongside the patterns for clarity. The sample codes (*e.g.*, 1 : 1.8, 650, 1 h, 16 h, 80, 24) represent the experimental conditions used for each sample: bentonite/NaOH ratio, fusion temperature (°C), fusion time (h), stirring time (h), crystallization temperature (°C), and crystallization time (h), respectively.

Fig. 3a shows the XRD spectra of samples that closely match the 4A phase and commercial Zeolite 4A reference. Characteristic peaks appear at around 2 theta ≈ 12.5°, 21.7°, 27°, 34° and 49°, are consistent with the LTA-type zeolite framework. The match in peak positions and relative intensities confirms the successful synthesis of Zeolite 4A. Furthermore, the result confirms that alkaline fusion at 500 °C followed by hydrothermal treatment at 100 °C for 8–24 h is effective for Zeolite 4A synthesis from bentonite clay. Fig. 3b, shows the successful synthesis of Zeolite 13X in which the main peaks appeared at 2 theta ≈ 6.2, 10, 15.8, 23.8, 30.8 and 31.6 are consistent with the FAU type structure of Zeolite 13X. The samples in Fig. 3c exhibit Zeolite Y type peaks, matching well with the commercial reference pattern. The appearance of the peaks similar to Zeolite 13X (due to shared FAU topology) is accompanied by shifts in peak intensities, suggesting a framework strain due to differences in Si/Al ratios.^62^ Samples prepared with higher NaOH ratios (≥1 : 1.8) and longer crystallization durations (up to 24 h) showed improved peak sharpness, supporting the formation of highly crystallized and pure FAU type zeolites.

These XRD results confirm that zeolite phase selectivity is governed by the alkaline fusion and crystallization conditions. Increasing fusion temperature and NaOH content promotes enhanced silica dissolution, shifting the effective Si/Al ratio of the synthesis gel and favoring FAU-type zeolites over LTA-type phases. This trend is consistent with the mechanistic pathway proposed in Fig. 1 and 9.

Among the multiple synthesized samples of each zeolite type, the samples exhibiting the highest phase purity and crystallinity, as confirmed by XRD, were selected to serve as representative materials for further characterization and performance evaluation. Phase purity was determined through XRD by comparing the diffraction patterns of the synthesized zeolite samples with those of corresponding commercial references and the IZA Structure database (International Zeolite Association). Samples exhibiting complete peak profiles and with no secondary peaks or elevated amorphous background were considered phase pure. The relative crystallinity of the selected sample from the Zeolite 4A categories was the sample with the synthesis conditions 1 : 1, 500 °C, 1 h, 24 h, 100 °C, 8 h, exhibiting the highest phase purity and relative crystallinity (approximately 117%) was selected as the representative of synthesized Zeolite 4A. Similarly, the sample with conditions 1 : 1.8, 650 °C, 1 h, 16 h, 80 °C, 8 h was selected as the representative synthesized Zeolite 13X with approximately 99% relative crystallinity; and the sample with conditions 1.8, 650 °C, 6 h, 24 h, 100 °C, 24 h was selected as the representative synthesized Zeolite Y with approximately 91% relative crystallinity. The integrated peak areas and calculated relative crystallinity for all samples within each zeolite category are provided in the SI Table 1.

**Fig. 4:**
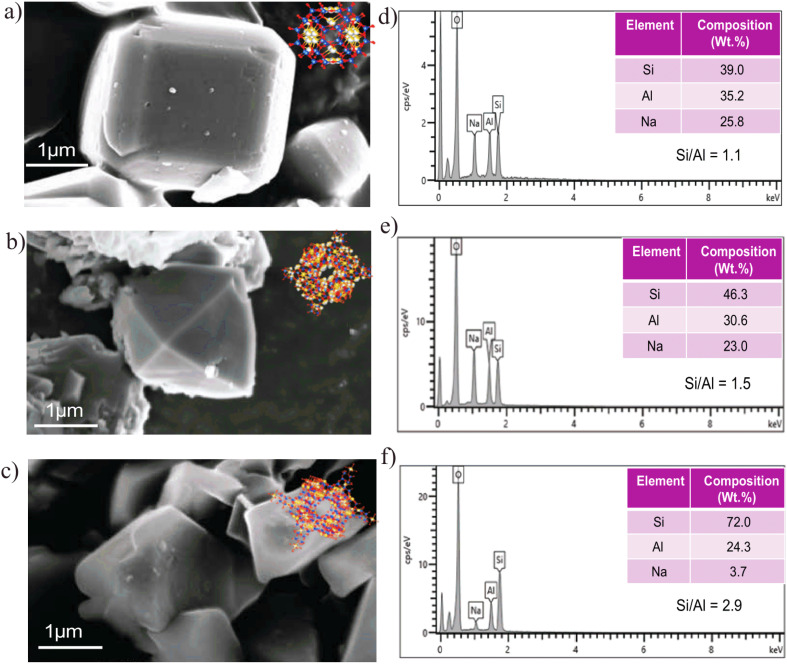
(a–c) SEM images of synthesized Zeolite 4A (a), Zeolite 13X (b), and Zeolite Y (c), (d–f) EDXS spectra and elemental compositions of the corresponding zeolites.

The obtained SEM images (a–c) show distinct crystal morphologies for each zeolite type. Zeolite 4A Fig. 4a exhibits well-defined cubic crystals with smooth surfaces and sharp edges, characteristic of its cubic framework structure. In contrast, Zeolite 13X and Zeolite Y (Fig. 4b and c, respectively) showed octahedral crystal morphologies, as both zeolite types share the faujasite framework, which inherently favors octahedral growth. On the other hand, Zeolite Y showed a more elongated octahedral morphology compared to Zeolite 13X. This difference could be attributed to its higher Si/Al ratio, as the reduced aluminium content may alter surface charge distribution and promote anisotropic crystal growth. In high-silica Y-type zeolites, the uneven formation of D6R (double six-membered ring) and D4R (double four-membered ring) units is known to cause preferential growth along directions perpendicular to the pore systems, resulting in the observed elongated morphology. The corresponding EDXS analyses (d–f) further confirm the successful synthesis of the zeolite types through elemental composition. Zeolite 4A (Fig. 4d) shows the presence of silicon (Si), aluminum (Al), oxygen (O), and sodium (Na), with a Si/Al ratio close to 1.1, which is consistent with the typical stoichiometry of 4A-type zeolites.^63^ Zeolite 13X (Fig. 4e) showed a slightly higher Si/Al ratio of approximately 1.5, in agreement with the expected characteristics of X-type zeolites.^64^ Zeolite Y (Fig. 4f) demonstrates the highest Si/Al ratio among the three samples (2.9), indicating its high-silica faujasite structure.^64^ The progressive increase in the Si/Al ratio from Zeolite 4A to Zeolite Y, as determined by EDX, aligns well with the designed synthesis strategy and confirms that controlled silica enrichment during alkaline fusion and hydrothermal treatment governs framework composition and phase evolution. Additionally, the morphology and elemental composition of the natural bentonite clay precursor were analyzed by SEM-EDX and are provided in the SI (Fig. S1), confirming its aluminosilicate-dominated nature with minor non-framework impurities typical of natural clays, which are substantially reduced during zeolite synthesis through alkaline fusion, hydrothermal crystallization, and subsequent washing steps.

#### 3.1.1 N_2_ adsorption–desorption isotherms

**Fig. 5:**
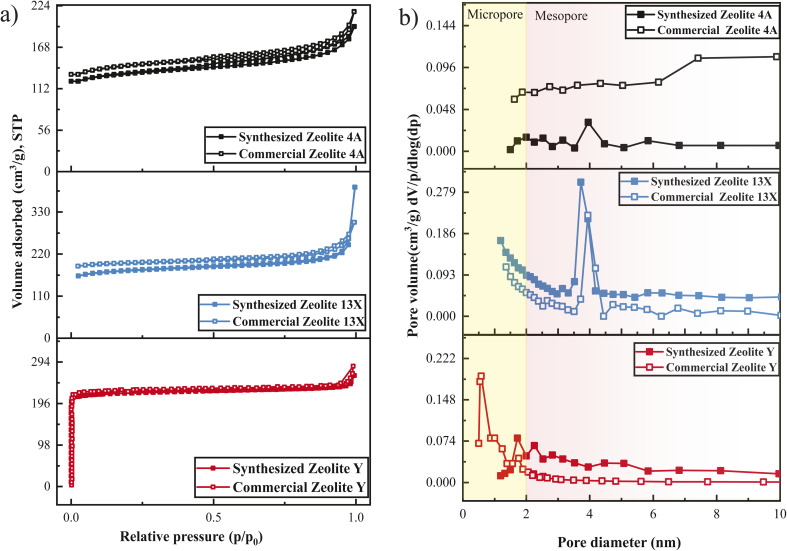
(a) Nitrogen adsorption–desorption isotherms at 77 K for synthesized Zeolite 4A, commercial Zeolite 4A, synthesized Zeolite 13X, commercial Zeolite 13X, synthesized Zeolite Y and commercial Zeolite Y. (b) BJH pore size distribution curves and average pore diameter.

The N_2_ adsorption–desorption isotherms of the synthesized Zeolite 4A, Zeolite 13X, and Zeolite Y are shown in Fig. 5a. According to IUPAC classification,^65^ the adsorption–desorption isotherm of Zeolite 4A showed type III behavior. In contrast, Zeolite 13X, and Zeolite Y primarily show Type I isotherms, which are characteristic of microporous materials. In the region *p*/*p*_0_ > 0.95, the isotherms began to increase sharply, indicating the presence of some textural mesopores. Among the synthesized samples, Zeolite 13X exhibited the highest N_2_ uptake at low relative pressures (*p*/*p*_0_ < 0.1), highlighting its microporous nature and the highest specific surface area. To further highlight the microporous adsorption behavior, an expanded view of the low relative pressure region (*p*/*p*_0_ ≤ 0.10) is provided in SI (Fig. S4). As shown in this magnified region, both the synthesized and commercial Zeolite 4A, Zeolite 13X, and Zeolite Y exhibit a pronounced and rapid uptake at very low relative pressures followed by early saturation, which is characteristic of micropore filling.^65^ The low-pressure adsorption trends of the synthesized zeolites closely match those of their corresponding commercial references, confirming comparable microporous behavior. The BET surface area and average pore diameter of synthesized Zeolite 13X were approximately 528 m^2^ g^−1^ (*p*/*p*_0_ = 0.075–0.300) and 2.79 nm, respectively. In comparison, synthesized Zeolite Y and synthesized Zeolite 4A showed BET surface areas of about 426 m^2^ g^−1^ (*p*/*p*_0_ ≈ 0.12–0.24) and 142 m^2^ g^−1^ (*p*/*p*_0_ = 0.076–0.299), with corresponding average pore diameters of 3.2 nm and 8.9 nm. The commercial samples followed a similar trend; commercial Zeolite 13X showed the highest surface area of 634 m^2^ g^−1^ (*p*/*p*_0_ = 0.076–0.300) with an average pore diameter of ∼2.8 nm, while commercial Zeolite Y, and Zeolite 4A showed BET surface areas of 449 m^2^ g^−1^ (*p*/*p*_0_ ≈ 0.12–0.24) and 202 m^2^ g^−1^ (*p*/*p*_0_ = 0.075–0.299), with average pore diameters of ∼2.8 nm and ∼8.9 nm, respectively. These results confirm the mesoporous dominated nature of Zeolite 4A and the presence of both microporous and mesoporous structures in Zeolite 13X and Zeolite Y. These textural differences are consistent with the phase-selective crystallization mechanism, where synthesis parameters determine framework topology and pore architecture through controlled silica–alumina speciation.

#### 3.1.2 CO_2_ adsorption performance evaluation

**Fig. 6:**
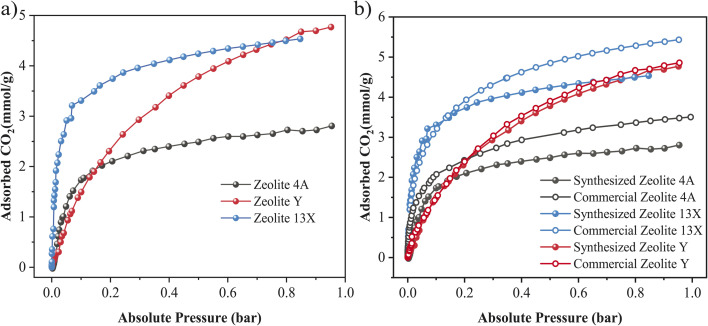
CO_2_ adsorption isotherms of synthesized and commercial zeolites at 25 °C. (a) Comparison of synthesized Zeolite 4A, Zeolite Y, and Zeolite 13X (b) comparison between synthesized and commercial counterparts of Zeolite 4A, Zeolite Y, and Zeolite 13X.

Fig. 6a presents the CO_2_ adsorption isotherms of the as-synthesized Zeolite 4A, Zeolite 13X, and Zeolite Y samples at 25 °C. Among the three, Zeolite 13X showed the highest CO_2_ uptake, reaching approximately 4.5 mmol g^−1^ at 1 bar, followed by Zeolite Y and Zeolite 4A. The higher CO_2_ adsorption performance of Zeolite 13X is attributed to its high BET surface area, and high microporosity.^23^ Zeolite Y also showed considerable adsorption capacity, being only slightly lower than Zeolite 13X, likely due to its higher Si/Al ratio and significantly reduced Na^+^ content (3.7 wt%, as shown in Fig. 4b, EDXS mapping), which reduces the number of available cationic sites.^3^ Zeolite 4A showed the lowest CO_2_ adsorption; this could be due to its low BET surface area and limited microporosity. Fig. 6b compares the CO_2_ adsorption performance of the synthesized zeolites with their corresponding commercial zeolite standards. Remarkably, the clay-based Zeolite 13X demonstrated a CO_2_ uptake capacity closely matching that of the commercial Zeolite 13X. In contrast, the synthesized Zeolite Y and Zeolite 4A showed slightly lower adsorption capacities compared to their commercial counterparts, likely due to differences in surface area, crystallinity, or cation distribution.^66^ The observed CO_2_ adsorption trends directly reflect the framework type and Si/Al ratio established during synthesis, with FAU-type Zeolite 13X providing a favorable balance of microporosity, surface area, and Na^+^ density, consistent with the phase-selective crystallization mechanism discussed earlier. Overall, these results indicate the potential of the clay-based synthesis approach to produce high performance zeolite adsorbents with competitive CO_2_ capture capacities despite using a significantly more environmentally benign process.

#### 3.1.3 Isosteric heat of adsorption measurements

**Fig. 7:**
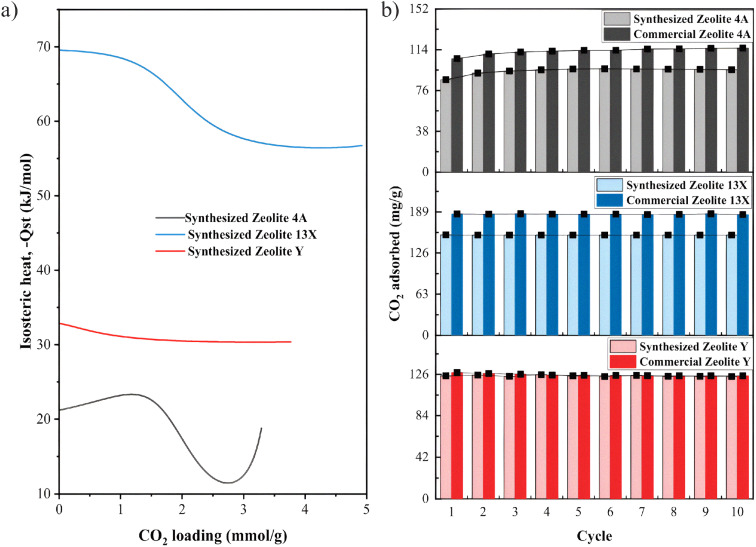
(a) Isosteric heat of CO_2_ adsorption (−*Q*_st_) as a function of CO_2_ loading for synthesized Zeolite 4A, synthesized Zeolite 13X, and synthesized Zeolite Y. Literature ranges for commercial Zeolite 4A, commercial Zeolite 13X an commercial Zeolite Y are summarized in Table S2 (see SI) for comparison (b) cyclic CO_2_ uptake of the synthesized zeolites (colored bars) compared with their commercial references (darker bars) over 10 adsorption–desorption cycles. Adsorption was performed at 25 °C and regeneration at 350 °C.

The isosteric heat of CO_2_ adsorption (−*Q*_st_) profiles of the synthesized zeolites are presented in Fig. 7a. The isotherms for each synthesized zeolites at three different temperatures (25–60 °C) and the dual site Langmuir fitting values are briefly presented in Table S8 (see SI). This measurement provides important information about the nature of the adsorption mechanism in the synthesized zeolites. Synthesized Zeolite 13X showed the highest initial −*Q*_st_ (≈68.8 kJ mol^−1^), suggesting very strong physisorption dominated by electrostatic interactions between CO_2_ molecules and Na^+^ cations.^30,67^ Although the interaction strength is closer to the upper limit of typical physisorption, the absence of chemical bond formation indicates that CO_2_ adsorption on Zeolite 13X remains a strong physisorption process rather than chemisorption. Synthesized Zeolite Y showed a moderate −*Q*_st_ (≈32.8 kJ mol^−1^), characteristic of typical physisorption interactions,^30^ while synthesized Zeolite 4A showed a relatively low and unstable −*Q*_st_ (≈19–22 kJ mol^−1^), reflecting weak physisorption likely due to low surface area and limited microporosity.^68^ Overall, the results confirm that the synthesized zeolites adsorb CO_2_ through physisorption mechanisms, with Zeolite 13X showing the most energetically favorable adsorption sites for efficient and reversible CO_2_ capture. This is consistent with observations made using commercial Zeolite 13X, Zeolite Y, and Zeolite 4A, which also rely on physisorption as the primary mechanism for CO_2_ uptake.^67,69^

#### 3.1.4 CO_2_ adsorption cyclic stability test

Evaluating the cyclic stability of the zeolite adsorbents is crucial to assess their regeneration capability and long-term performance. Therefore, a 10-cycle CO_2_ adsorption–desorption test was conducted for the synthesized and commercial Zeolite 4A, Zeolite 13X, and Zeolite Y samples. After each CO_2_ adsorption step (conducted at 25 °C and 1 bar), the adsorbents were regenerated by purging with N_2_ at 350 °C. As shown in Fig. 7b, across ten adsorption–desorption cycles, all samples retained their CO_2_ uptake with negligible loss. The synthesized Zeolite 13X maintained constant adsorption capacity at ∼153 mg g^−1^, while the commercial 13X showed ∼186 mg g^−1^. The synthesized Zeolite 4A sustained ∼90–100 mg g^−1^ compared with ∼112–118 mg g^−1^ for the commercial 4A. For Zeolite Y, the adsorption capacities were nearly unchanged over cycling, ∼122–124 mg g^−1^ for the synthesized sample *versus* ∼125–126 mg g^−1^ for the commercial reference. These results show the strong potential of the clay-derived zeolite adsorbents for repeated CO_2_ capture-regeneration operations without significant degradation, making them promising for practical carbon capture technologies. Among all tested samples, Zeolite 13X demonstrated the best combination of high CO_2_ uptake and cyclic stability.

Key properties of the synthesized materials relative to their commercial counterparts are summarized in Table 2. Commercial −*Q*_st_ values were given as literature ranges Table S3, (see SI).

**Table 2:** Key properties of the synthesized aeolites relative to their commercial counterparts^70–74^

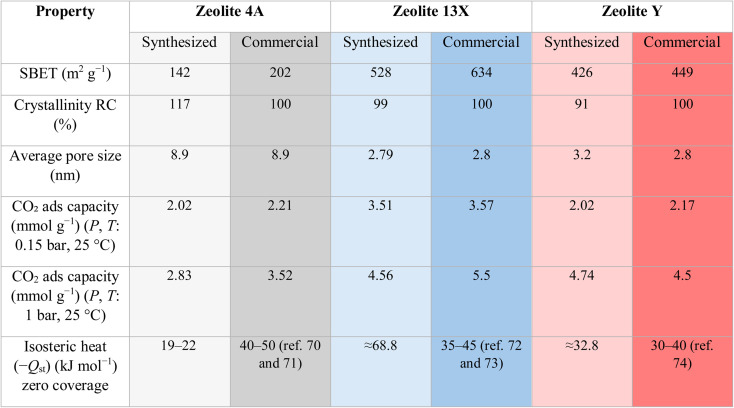

#### 3.1.5 Water adsorption performance evaluation

**Fig. 8:**
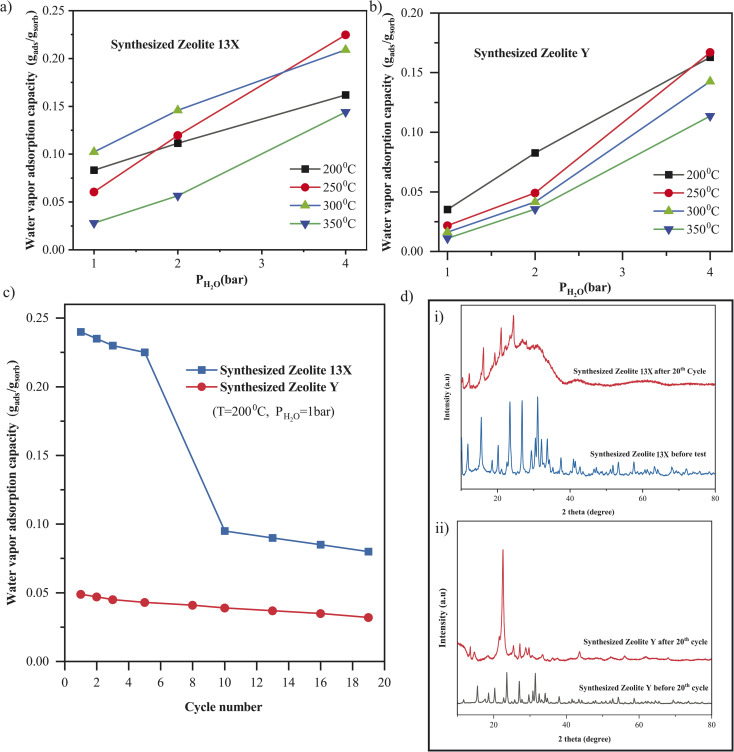
(a) Water vapor adsorption isotherms of synthesized Zeolite 13X at 200 °C, 250 °C, 300 °C, and 350 °C. (b) Water vapor adsorption isotherms of synthesized Zeolite Y under the same conditions. (c) Cyclic water vapor adsorption stability tests of synthesized Zeolite 13X and Zeolite Y at 200 °C and 1 bar. (d) (i) XRD patterns of synthesized Zeolite 13X before and after 20 water vapor adsorption–desorption cycles at 200 °C and 1 bar. The samples were reactivated between cycles (see (Method) (ii) XRD pattern of synthesized Zeolite Y after 20 cycles under identical conditions).

Water vapor adsorption plays a crucial role in many industrial applications, where zeolites are exposed to elevated temperatures and steam, including such applications as gas purification, drying processes, and catalytic systems.^75–77^ Zeolites such as 13X and Y are widely used in such environments.^78,79^ In addition, zeolites are increasingly used as catalyst supports in sorption-enhanced CO_2_ conversion processes, such as methanation or synthetic fuel production, where their intrinsic water adsorption capacity helps shift the reaction equilibrium toward product formation by removing water *in situ*; particularly under low-pressure conditions where reaction efficiency is otherwise thermodynamically limited.^80,81^ Therefore, evaluating the water vapor adsorption capacity and thermal stability of zeolites at elevated pressures and temperatures is essential to determine their practical viability in both adsorption and catalytic roles. Accordingly, the water vapor adsorption performance of both synthesized Zeolite 13X and Zeolite Y was evaluated at 200 °C, 250 °C, 300 °C, and 350 °C under varying water vapor pressures ranging from 1 to 4 bar (Fig. 8(a and b)). For Zeolite 13X (Fig. 8a), the adsorption capacity consistently increased with increasing water vapor pressure at all temperatures, indicating pressure-driven sorption behavior. The highest capacity (≈0.22 g_ads_ g_sor_^−1^) was recorded at 250 °C and 4 bar. A decline in adsorption capacity was observed with rising temperature, with 350 °C showing the lowest uptake (≈0.13 g_ads_ g_sor_^−1^ at 4 bar), consistent with the exothermic nature of water adsorption.^82^ For Zeolite Y (Fig. 8b), a similar increase in adsorption capacity with pressure was observed. However, Zeolite Y exhibited generally lower adsorption capacities than Zeolite 13X across all tested conditions. The maximum adsorption (∼0.16 g_ads_ g_sor_^−1^) was achieved at 250 °C and 4 bar, with a less steep decline at higher temperatures compared to Zeolite 13X. The difference in adsorption performance between the two zeolites can be attributed to their Si/Al ratios. Zeolite 13X, with a lower Si/Al ratio of ≈1.5, has a higher aluminum content, resulting in more negatively charged framework sites and, consequently, more hydrophilic character.^61,83^ This enhances its affinity for polar water molecules, leading to higher adsorption capacity. In contrast, Zeolite Y has a higher Si/Al ratio of ∼2.9, making it relatively more hydrophobic and less effective in water vapor adsorption.^84^ Thus, Zeolite 13X outperforms Zeolite Y in water vapor uptake under all conditions tested, particularly at moderate temperatures (250–300 °C), due to its higher hydrophilicity and stronger interaction with water molecules.

The cyclic stability of water vapor adsorption was further investigated for both synthesized Zeolite 13X and Zeolite Y under hydrothermal conditions of 200 °C and 1 bar water vapor pressure (Fig. 8c). For Zeolite 13X, a significant decrease in adsorption capacity was observed over 19 cycles, dropping from ≈0.24 g_ads_ g_sor_^−1^ in the first cycle to below 0.1 g_ads_ g_sor_^−1^, indicating rapid performance degradation. This behavior is attributed to hydrothermal instability, due to framework degradation, pore collapse, or dealumination under repeated exposure to humid heat (Fig. 8d(i)). In contrast, Zeolite Y displayed a more stable performance under identical conditions, with only a gradual decline in capacity (≈0.05 to ≈0.03 g_ads_ g_sor_^−1^) over the same number of cycles. The improved stability of Zeolite Y is likely linked to its higher Si/Al ratio (≈2.9), which enhances its resistance to hydrothermal dealumination^85^ compared to the more aluminum-rich Zeolite 13X (Si/Al ≈ 1.5). These performance trends are further supported by structural XRD analysis Fig. 8d(i), which reveals significant framework degradation in Zeolite 13X after cycling, whereas Zeolite Y retains its structural integrity better than Zeolite 13X (Fig. 8d(ii)). While Zeolite 13X showed higher initial water adsorption capacity, its limited stability under repeated cycling highlights the need for structural reinforcement or alternative optimization strategies for long-term sorption applications. We note that these observations match those reported in the literature for commercial zeolites of the same types.^86,87^ These trends observed in synthesized Zeolite 13X and Zeolite Y directly reflect the synthesis-controlled framework composition, whereby selective silica–alumina dissolution during alkaline fusion and subsequent crystallization governs both the hydrophilicity and hydrothermal stability of the resulting zeolites.

#### 3.1.6 Effect of synthesis parameters on the zeolite formation mechanism

In this study, bentonite clay is used as the sole source of silicon and aluminum for zeolite synthesis. While the bulk Si/Al ratio of bentonite defines the total availability of silicon and aluminum, the effective Si/Al ratio of the synthesis gel and thus of the resulting zeolite structure is governed by selective dissolution and reorganization of aluminosilicate species during alkaline fusion and subsequent hydrothermal treatment. These processes are strongly controlled by synthesis parameters, including the bentonite/NaOH ratio, fusion temperature, and crystallization conditions, rather than by the bulk clay composition alone.

**Fig. 9:**
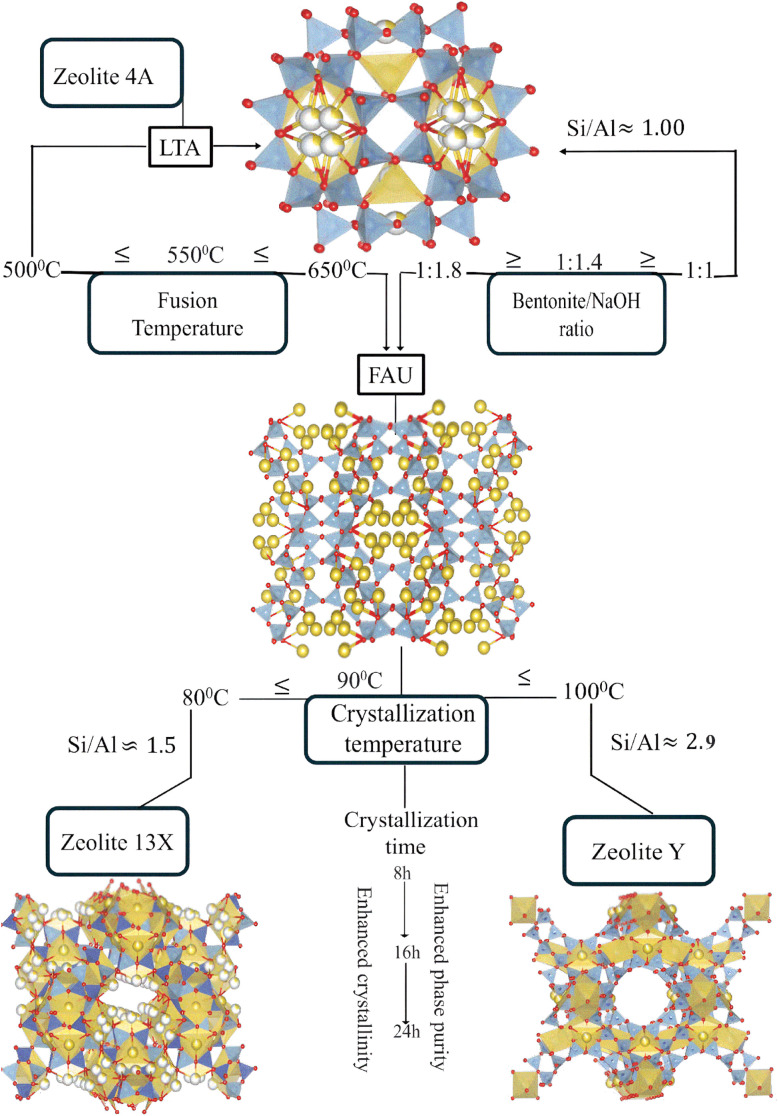
Mechanistic representation of zeolite phase formation from bentonite clay.

The mechanistic role of these synthesis parameters in directing phase-selective crystallization is summarized in Fig. 9 and supported by results. The schematic illustrates the molecular transformation pathways of bentonite clay evolves into distinct zeolite frameworks as a function of systematic tuned synthesis parameters. Upon alkaline fusion with sodium hydroxide (NaOH), the layered aluminosilicate structure of montmorillonite undergoes depolymerization, breaking down into reactive Si and Al species.^25,58,88^ The extent of this depolymerization and the relative availability of silica *versus* alumina are strongly governed by the fusion temperature and the bentonite/NaOH weight ratio. At lower fusion temperatures (≤500 °C) and at a bentonite/NaOH ratio of ≈1 : 1, the reaction medium becomes alumina-rich, favoring the crystallization of low-silica zeolites. Under these conditions, Zeolite 4A with a characteristic LTA framework (Si/Al ≈ 1) is formed.^59,60^ In contrast, at high fusion temperature (>550 °C) and as the bentonite/NaOH ratio increases (≥1 : 1.4), the system facilitates greater silica dissolution, shifting the composition toward a silica-rich environment. This change promotes the formation of FAU type zeolites, such as Zeolite 13X (Si/Al ≈ 1.5) and Zeolite Y (Si/Al ≈ 2.9), depending on crystallization conditions.^23,61^ The hydrothermal crystallization stage further directs the structural evolution of the product. Lower crystallization temperatures (≈80 °C) favor Zeolite 13X, while higher temperatures (≈100 °C) and extended durations promote the condensation of silica rich species, facilitating the formation of Zeolite Y. This temperature and time dependent crystallization behavior reflects the increased thermodynamic stability required for forming high-silica frameworks. Overall, this mechanistic framework highlights how targeted adjustments to fusion and crystallization conditions enable the controlled synthesis of structurally diverse zeolites from a single clay precursor.

While this work focuses solely on bentonite clay, we note that the synthesis strategy demonstrated in this work is not limited to bentonite clay but can be extended to a wide range of naturally occurring aluminosilicate clays, such as kaolinite, illite, and mixed layer clay minerals. The key requirement is the presence of reactive Si and Al species that can be liberated through alkaline activation or fusion. While the bulk Si/Al ratio and impurity content of different clays may vary, the present results show that selective dissolution and reorganization of aluminosilicate species during alkaline fusion and hydrothermal treatment govern zeolite phase formation, rather than the initial clay composition alone.

Accordingly, by adjusting synthesis parameters such as alkali concentration, fusion temperature, and crystallization conditions, different zeolite frameworks can be targeted even when using clays with distinct mineralogical compositions. Previous studies have reported successful zeolite synthesis from various natural clays using similar activation–recrystallization approaches, supporting the broader applicability of this strategy.^89,90^ Therefore, the methodology presented here provides a flexible and scalable route for converting diverse low-cost clay resources into value added zeolite materials.

### 3.2. LCA comparison

The total environmental impact of both chemical and clay based (this work) synthesis systems were calculated and are analyzed in detail below.

#### 3.2.1 Global warming potential and cumulative energy demand

**Fig. 10:**
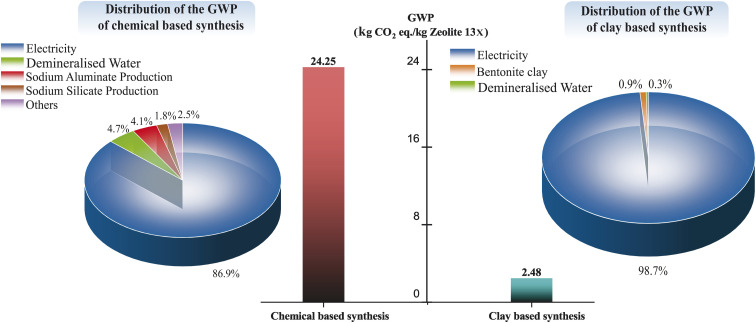
Comparison and distribution of the GWP of the chemical and clay-based zeolite synthesis.

**Fig. 11:**
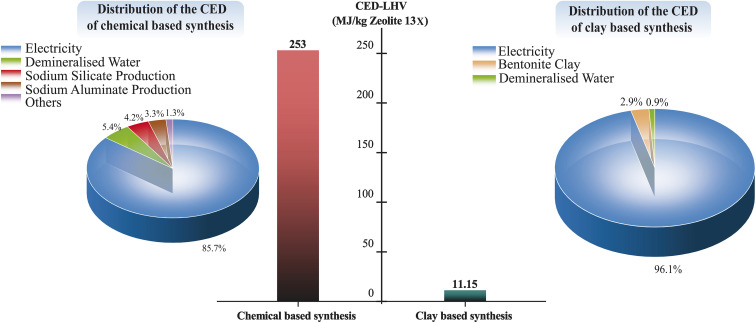
Comparison and distribution of the CED-LHV of the chemical and clay-based zeolite synthesis.

Conventional zeolite production, which typically relies on chemical-based synthesis routes, is highly energy-intensive and associated with a significant carbon footprint.^6,91^ Given these characteristics, evaluating the Global Warming Potential (GWP) and Cumulative Energy Demand (CED) is essential for understanding the environmental impacts of zeolite production. Fig. 10 and 11 show a comparison of the GWP and CED and their distributions for chemical-based and clay-based synthesis of Zeolite 13X. As shown in Fig. 10, the carbon footprint of Zeolite 13X synthesized *via* the chemical-based route is 24.25 kg CO_2_-eq per kilogram of product, which is almost 10 times higher than that of the clay-based synthesis route (2.48 kg CO_2_-eq per kilogram of Zeolite 13X). The analysis further indicates that electricity consumption is the dominant contributor to the carbon footprint in both synthesis methods, accounting for 86.9% of the total impact in the chemical-based process and 98.7% in the clay-based process. In the clay-based zeolite synthesis route, electricity consumption is primarily associated with the activation of bentonite clay and the hydrothermal crystallization process. In contrast, in the chemical-based system, electricity consumption results from the cumulative energy demands of multiple steps, including the production of silicon and aluminum sources, raw material activation, and the hydrothermal crystallization process. Regarding the raw material GWP contribution, the production of bentonite clay involves relatively simple processes, including mining, processing, and activation in clay-based Zeolite 13X synthesis mechanism. In contrast, in the chemical-based synthesis route, the production of raw materials for silicon and aluminum sources can be the major contributors to the overall GWP. In the production of sodium aluminate, the pre-dominant method involves extracting sodium aluminate from bauxite *via* Bayer process.^92^ Following mining, grinding, and crushing, the bauxite ore is digested in a sodium hydroxide solution under high-temperature and high-pressure conditions, yielding a sodium aluminate solution and an insoluble by-product known as red mud.^93^ Red mud, composed primarily of iron, silicon, and titanium oxides and is classified as a hazardous waste due to its strong corrosivity and significant risks to human health and the environment.^94^ Similarly, the production of sodium silicate requires the mining and purification of quartz sand (to remove alumina and iron oxide impurities), followed by high-temperature melting of soda ash (>1400 °C), and subsequent dissolution, filtration, and concentration steps to produce solid sodium silicate.^6,44^ The complexity and high energy demands associated with the preparation of these chemical precursors contribute to the high Global Warming Potential (GWP) observed for chemical based synthesized zeolites. It is worth noting that sodium hydroxide (NaOH) is used in both the chemical-based and clay-based zeolite synthesis routes. However, its individual contribution to the overall Global Warming Potential (GWP) is below the reporting threshold in both cases.

Fig. 11 illustrates that the energy consumption of the chemical-based Zeolite 13X synthesis route (253 MJ per kilogram of Zeolite 13X) is approximately 22 times higher than that of the clay-based synthesis route (11.15 MJ per kilogram of Zeolite 13X). A trend similar to the GWP contributors is observed in the CED contributors. In both synthesis routes, electricity consumption remains the dominant factor, accounting for 85.7% of the total energy demand in the chemical-based process and 96.1% in the clay-based process. For the chemical-based synthesis route, the next highest contributors to cumulative energy demand are demineralized water production (5.4%), sodium silicate production (4.2%), and sodium aluminate production (3.3%). In the case of the clay-based Zeolite 13X synthesis, bentonite clay preparation (2.9%) shows the subsequent highest contributors to overall energy consumption.

#### 3.2.2 Environmental toxicity profile

**Fig. 12:**
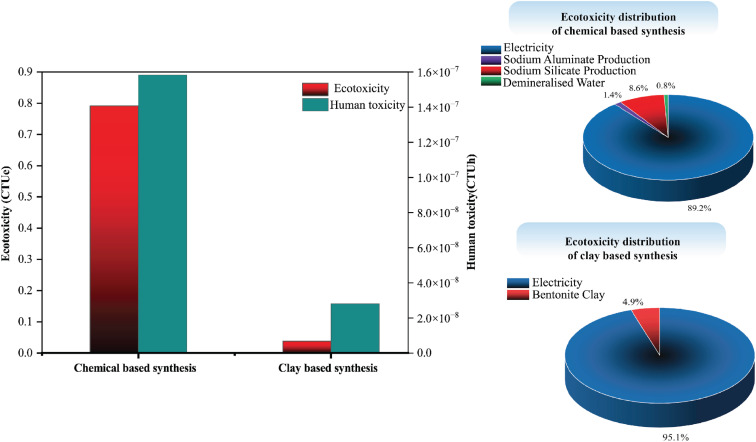
Environmental profile comparison and ecotoxicity distribution of the CED-LHV of the chemical and clay-based zeolite synthesis.

Fig. 12, shows a comparison of ecotoxicity and human toxicity between the chemical-based and clay-based synthesis routes for Zeolite 13X production. Ecotoxicity and human toxicity impacts were assessed using Comparative Toxic Units for ecosystems (CTUe) and Comparative Toxic Units for humans (CTUh), respectively, based on opeLCA environmental impact assessment method. Based on the analysis results, the chemical-based synthesis route shows significantly higher values for both ecotoxicity (≈20 times higher) and human toxicity (≈5.6 times higher) than that from clay-based synthesis route. The main reason for this lies in the production of raw materials like the silicon and aluminum source. In the chemical-based route, electricity consumption is the dominant contributor to both ecotoxicity (0.72 CTUe) and human toxicity (≈5.5 × 10^−7^ CTUh). Sodium aluminate production contributes significantly to human toxicity (≈0.73 × 10^−7^ CTUh) but negligibly to ecotoxicity. In contrast, in the clay-based synthesis route, the ecotoxicity and human toxicity values associated with electricity are significantly lower (≈0.037 CTUe and ≈2.0 × 10^−8^ CTUh, respectively), and the contribution from bentonite clay preparation remains minimal.

The impact assessment methods employed clearly demonstrate that the clay-based approach proposed herein significantly reduces energy consumption, negative environmental impacts, and associated emissions. Therefore, the clay-based synthesis route presents a more sustainable and environmentally favorable alternative for Zeolite 13X production. Additionally, although the LCA comparison in this study was conducted specifically for Zeolite 13X, all synthesized zeolites in this work follow a similar synthesis mechanism. Given the sustainable results observed for Zeolite 13X, it can be reasonably concluded that the clay-based approach offers a more sustainable and environmentally favorable route for zeolite synthesis in general.

### 3.3. Cost-oriented economic comparison

**Fig. 13:**
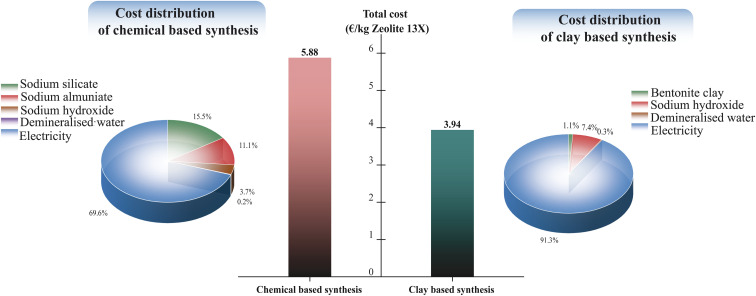
Cost-oriented economic comparison of chemical based and clay-based synthesis routes.

Fig. 13 compares the total production cost of Zeolite 13X *via* chemical based and clay-based synthesis routes, normalized to 1 kg of Zeolite 13X product. The total cost values shown in Fig. 13 were calculated by combining LCA-derived material and energy inventories with representative unit prices, with detailed cost breakdowns provided in the SI (Tables S9 and S10). The clay-based route showed a substantially lower total cost (3.94 € per kg) compared to the chemical-based route (5.88 € per kg), corresponding to an approximate 33% cost reduction. Cost distribution analysis indicates that electricity consumption dominates both synthesis routes; however, the chemical-based synthesis shows significantly higher contributions from precursor materials, particularly sodium silicate and sodium aluminate. In contrast, the clay-based route benefits from the use of a low-cost natural raw material, resulting in reduced material related costs. This trend is consistent with the LCA results, where energy consumption was identified as the primary contributor to the overall environmental impacts.

## 4 Conclusion

This work demonstrates the feasibility of producing structurally and functionally diverse zeolites from a single natural clay precursor through a unified pathway. By understanding and exploiting systematic control over fusion and crystallization parameters, we established a mechanistic basis for selectively obtaining Zeolite 4A, Zeolite 13X, or Zeolite Y without synthetic reagents. The comparable performance of the synthesized zeolites to commercial benchmarks validates the robustness of this approach for adsorption-based applications. Beyond materials performance, the life cycle assessment and cost oriented economic analysis of the synthesis route reveal compelling environmental and economic advantages, highlighting its potential as a scalable and low-impact alternative to conventional zeolite production. The demonstrated structure–property–sustainability link underscores the broader applicability of this method across material classes where phase selectivity, functionality, and environmental impact must be balanced. Future work could explore extending this framework to other clay types, tuning different zeolite structures for specific separations, and integrating this synthesis route into circular resource loops.

## Author contributions

B. A. L. contributed to the conceptualization of the study, material synthesis, experimental characterizations, and data interpretation. V. S., as the principal investigator, contributed to the study's conceptual framework, methodological design, experimental validation, data analysis, supervision, and acquisition of research funding. B. A. L. and V. S. jointly wrote and edited the manuscript. W. L. Q. provided access to laboratory facilities, supervised some experimental work, and contributed to manuscript revision. J. E. and P. R. contributed to the CO_2_ adsorption experiments, heat of adsorption measurements, and surface characterizations carried out at LFIM. F. K. conducted the water vapor sorption experiments at Empa and contributed to the analysis of the resulting data. All authors reviewed and approved the final version of the manuscript.

## Conflicts of interest

The authors declare no competing interests.

## Supplementary Material

SE-010-D5SE01375E-s001

## Data Availability

The data supporting the findings of this study are available from the corresponding author upon reasonable request. All relevant figures and supplementary information are provided within the manuscript and the supplementary information (SI) file. Supplementary information: additional experimental details, extended characterization data, supporting figures, and tables referenced in the manuscript. See DOI: https://doi.org/10.1039/d5se01375e.
