# Supplementary material for: A sustainable multi-zeolite synthetic framework from a single natural clay: CO2/H2O adsorption performance and life cycle assessment benefits
Source: Sustain Energy Fuels. 2026 Jan 19;10(4):1038–58. doi: 10.1039/d5se01375e (PMC12826357; doi:10.1039/d5se01375e)
Supplement: SE-010-D5SE01375E-s001 [file SE-010-D5SE01375E-s001.pdf]

Supplementary Information (SI) for Sustainable Energy & Fuels.
This journal is © The Royal Society of Chemistry 2026

A Sustainable Multi-Zeolite Synthetic Framework from a Single Natural Clay: CO_2/H_2O Adsorption Performance and Life Cycle Assessment Benefits

Biruktait Ayele Lemecho[1]*, Jordi Espín[2], Pattaraphon Rodlamul[2], Florian Kiefer[3] , Wendy Lee Queen[2]*, Vivek Subramanian[1]

[1]Laboratory for Advanced Fabrication Technologies (LAFT), Institute of Electrical and Micro Engineering, Ecole Polytechnique Fédérale de Lausanne (EPFL), Neuchâtel 2000, Switzerland

[2] Laboratory for Functional Inorganic Materials (LFIM), Institut des Sciences et Ingénierie Chimiques, École Polytechnique Fédérale de Lausanne (EPFL), Rue de l'Industrie 17, 1951, Sion, Switzerland

[3] Chemical Energy Carriers and Vehicle Systems Laboratory Empa - Swiss Federal Laboratories for Materials Science and Technology 8600 Dübendorf, Switzerland

Supplementary information

Figure S1. SEM image of the natural bentonite clay precursor showing its characteristic layered, plate-like morphology, together with the corresponding EDX spectrum and elemental composition.

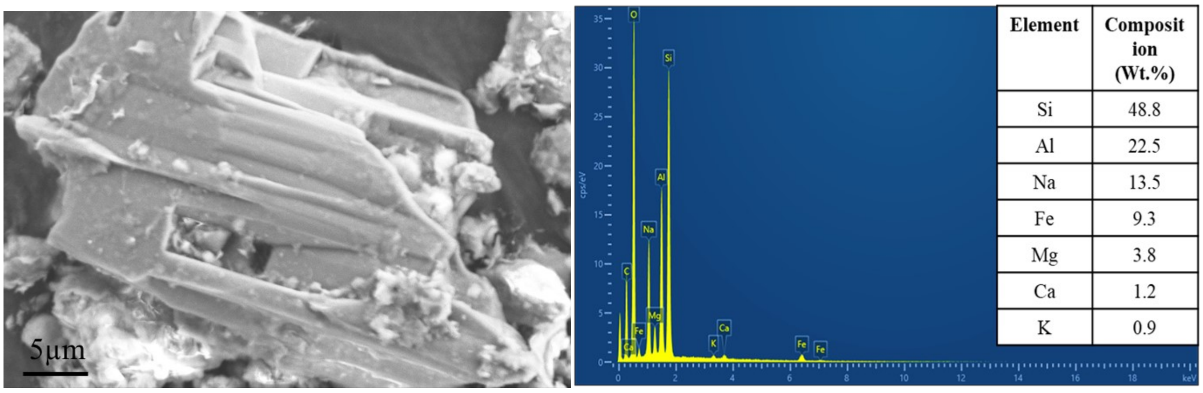

Element	Composition (Wt.%)
Si	48.8
Al	22.5
Na	13.5
Fe	9.3
Mg	3.8
Ca	1.2
K	0.9

Figure S2. Powder X-ray diffraction (PXRD) data of bentonite clay and high-angle region of Zeolites. (a) PXRD of the starting bentonite clay, normalized to the most intense peak. (b-d) High-angle zoom (50-100° 2θ) of normalized PXRD patterns for the synthesized zeolites and their commercial references (b) Zeolite 4A (LTA), (c) Zeolite 13X (FAU-X), (d) Zeolite Y (FAU-Y).

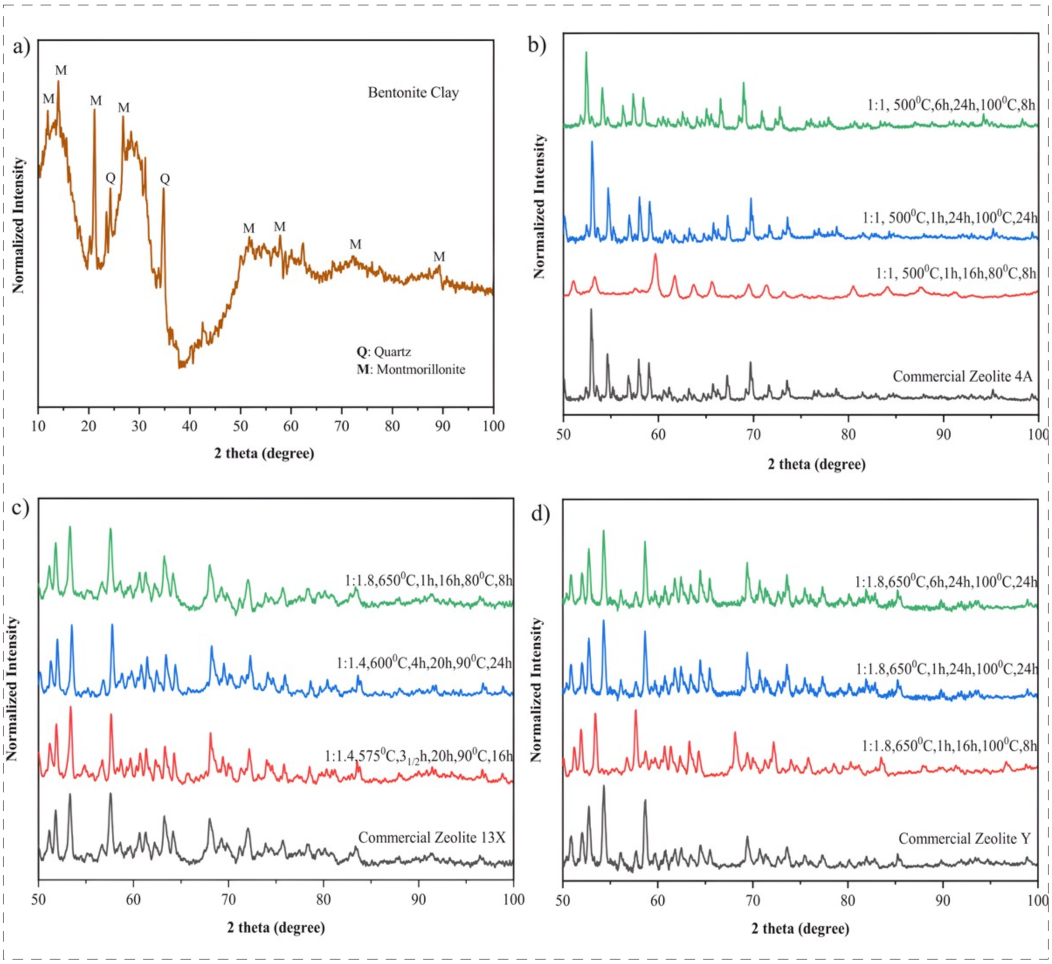

Table S1: Integrated peak areas and calculated relative crystallinity of all samples within each zeolite category

No	Zeolite type	Sample code	∑Synthesized zeolite	∑Commercial	Relative crystallinity (%)
1.	Zeolite 4A	1:1,500^{0}C,1h,16h,80^{0}C,8h	34028.53	39057.68	87.12
		1:1,500^{0}C,1h,24h,100^{0}C,24h	14619.87		37.43
		1:1, 500^{0}C,6h,24h,100^{0}C,8h	45835.33		117.35
2.	Zeolite 13X	1:1.4,575^{0}C,31/2h,20h,90^{0}C,16h	23742.16	26038.94	91.18
		1:1.4,600^{0}C,4h,20h,90^{0}C,24h	25051.65		96.21
		1:1.8,650^{0}C,1h,16h,80^{0}C,8h	25812.63		99.13
3.	Zeolite Y	1:1.8,650^{0}C,1h,16h,100^{0}C,8h	28127.11	56985.11	49.36
		1:1.8,650^{0}C,1h,24h,100^{0}C,24h	49604.34		87.05
		1:1.8,650^{0}C,6h,24h,100^{0}C,24h	51763.74		90.83731

Table S2: Literature ranges of -Q_{st} for commercial Zeolites

Zeolite (commercial)	−Qst (kJ mol⁻¹)	Typical T set (K)	Used method	References
Na-4A (Zeolite 4A)	40-50	273-333	Clausius-Clapeyron	[1,2]
Na-13X (Zeolite 13X)	35-45	288-333	Clausius-Clapeyron	[3,4]
Na-Y (Zeolite Y)	30-40	303-473	Clausius-Clapeyron	[5]

Figure S3: Schematic of the water-vapour adsorption setup.

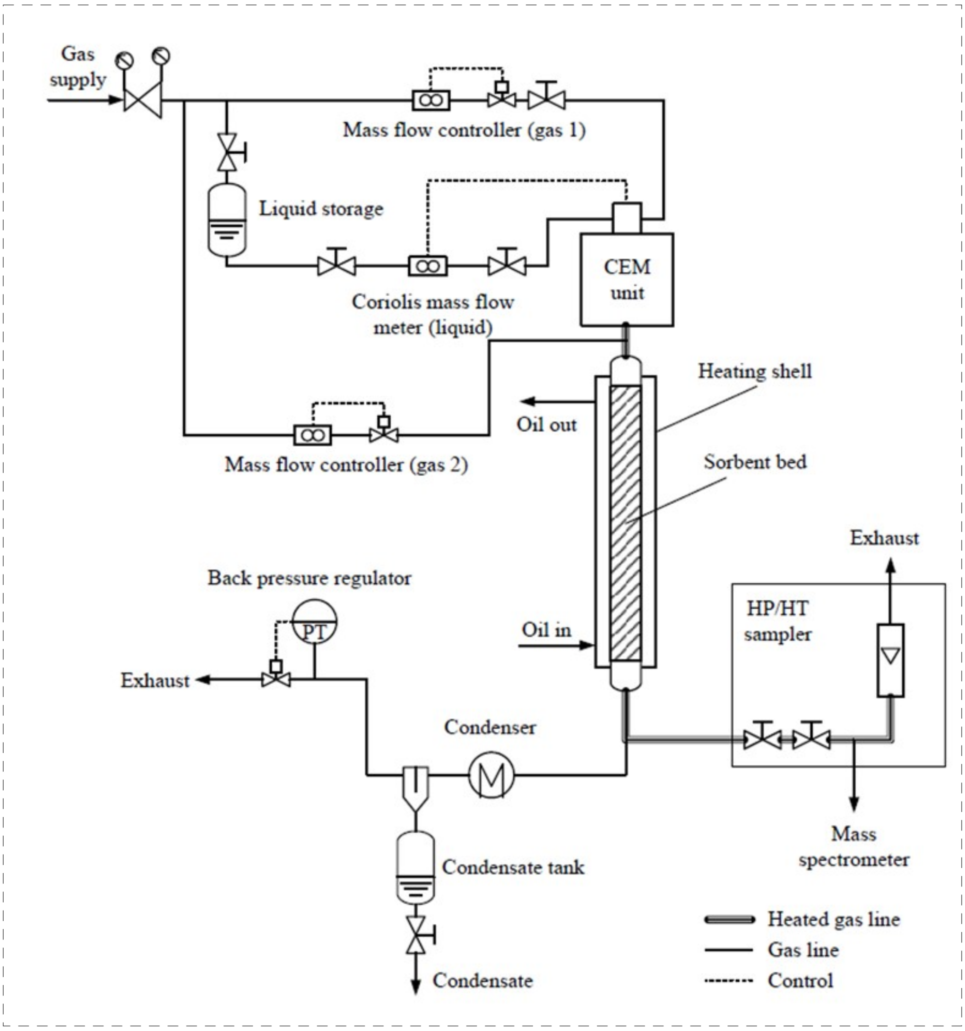

Table S3: Water sorption isotherms experiment summary for Zeolite 13x

Experiment	T(^{0}C)	P (bar)	P_{H2O} (bar)	Water breakthrough	Capacity_ g $_{ads}$/g_{sorb}	Asymmetry	R^2
Cycle-1	200	10	1	454.0797	0.245	12.3666	0.99755
Cycle-2	200	10	1	440.7093	0.23223	14.2052	0.99918
Cycle-3	200	10	1	433.763	0.23069	12.0599	0.9986
Cycle-4	200	10	1	431.5597	0.23889	11.6919	0.9975
Cycle-5	200	10	1	431.7157	0.23226	12.3833	0.9982
Cycle-1	300	10	0.2	158.1974	0.090942	41.7925	0.99914
Cycle-2	300	10	1	171.6267	0.10229	10.993	0.99687
Cycle-3	300	10	2	140.8708	0.14589	0.13133	0.98777
Cycle-4	300	10	4	67.5509	0.209	7.8738	0.98974
Cycle-5	200	10	1	153.4941	0.097125	15.6047	0.99729
Cycle-1	250	10	1	89.433	0.060627	6.6705	0.99605
Cycle-2	250	10	2	91.3596	0.11961	11.5258	0.98526
Cycle-3	250	10	4	58.4256	0.22475	6.6953	0.97692
Cycle-4	200	10	1	137.2601	0.083252	11.0361	0.99755
Cycle-1	200	10	2	77.77935	0.11145	3.3251	0.9752
Cycle-2	200	10	4	52.44405	0.16185	8.1972	0.9734
Cycle-3	200	10	1	122.0814	0.074027	9.0471	0.99714
Cycle-1	350	10	1	40.2686	0.028051	27.9609	0.9886
Cycle-2	350	10	2	44.882	0.056479	2274206	0.98531
Cycle-3	350	10	4	25.4186	0.14413	19.473	0.9982

Table 3 : Water sorption isotherms experiment summary for Zeolite Y

Experiment	T (^0C)	P (bar)	P_{H2O} (bar)	Water breakthrough	Capacity g_{ads}/g_{sorb}	Asymmetry	R^2
cycle_1	200	10	1	55.1944	0.044952	6.648	0.99233
cycle_2	200	10	1	52.237	0.042793	6.1018	0.99277
cycle_3	200	10	1	62.5122	0.044937	9.8074	0.99034
cycle_4	200	10	1	61.181	0.044515	11.2117	0.99121
cycle_1	300	10	1	35.4	0.01615	3.1619	0.99981
cycle_2	300	10	2	44.8306	0.041515	2913914	0.94738
cycle_3	300	10	4	46.4234	0.14262	10.6098	0.98058
cycle_4	200	10	1	65.4551	0.040156	71.0653	0.99184
cycle_1	250	10	1	41.6704	0.021732	34.8541	0.99374
cycle_2	250	10	2	62.7583	0.04916	727115.4	0.8199
cycle_3	250	10	4	80.7054	0.16694	68.4583	0.97101
cycle_4	200	10	1	60.965	0.041882	12.1009	0.99253
cycle_1	200	10	1	61.0004	0.035197	34.6106	0.98256
cycle_2	200	10	2	56.7048	0.082596	6.46	0.97332
cycle_3	350	10	1	24.34	0.0111	41.8146	0.99999
cycle_4	350	10	2	37.3566	0.035542	3983026	0.88235
cycle_5	350	10	4	16.4687	0.11368	3.8097	0.9911
cycle_6	200	10	1	56.6759	0.03255	36.4957	0.98924
cycle_1	300	10	1	31.1059	0.022459	435486.7	0.95641
cycle_2	200	10	4	74.8009	0.16284	0.40687	0.95959

Table 4: Input and output inventories to produce 1kg of Zeolite 13X from Bentonite clay.

	**Category**	**Unit**	**Value**	**Database for provider source**
Input	Bentonite Clay	kg	0.417	Ecoinvent 3.1
	Sodium hydroxide	kg	0.583	OZLCI2019
	Demineralized water	m3	0.0096	OZLCI2019
	Electricity	MJ	108.00	Electricity Mix-CH

Output	Bentonite clay-Zeolite 13x	Kg	1.00	This Work
Emission	Wastewater	m3	0.0094	This work, Ecoinvent 3.1

Table 5: Input and output inventories to produce 1kg of Zeolite 13X from chemical precursors. Reference work: International Intellectual Property : WO 2023/119309[6]

	**Category**	**Unit**	**Value**	**Database source for Provider**
Input	Sodium Silicate	Kg	1.517	Ecoinvent 3.1<br>USEEI , OZLCI2019
	Sodium Aluminate	Kg	0.540	Ecoinvent 3.1<br>OZLCI2019
	Sodium hydroxide	Kg	0.443	OZLCI2019
	Demineralized water	m3	0.00878	OZLCI2019
	Electricity	MJ	122.760	Electricity Mix-EU-27
Output	Chemical-Zeolite 13X	Kg	1.00	Patent (WO 2023/119309 AI)[6]
Emission	Wastewater	m3	0.00852	Patent (WO 2023/119309 AI)[6]

Table 6: Input and output inventories for 1kg sodium silicate production

	Category	Unit	Value	Database source for Provider
Input	Ground Quartzite	Kg	0.772	Ecoinvent 3.1, OZLCI2019, Reference[7]
	Fine Sand	Kg	0.65	Ecoinvent 3.1 , OZLCI2019, Reference [7]
	Crashed limestone	Kg	0.022	Ecoinvent 3.1 , OZLCI2019, Reference[7]
	Sodium hydroxide	Kg	0.209	OZLCI2019, Reference[7]
	Sodium chloride	kg	0.123	Ecoinvent 3.1 , OZLCI2019, Reference[7]
	Demineralized water	m3	0.8288	OZLCI2019, Reference [7]
	Electricity	MJ	3.118	Electricity grid mix, AC, < 1kV
	Diesel	Kg	0.144	Ecoinvent 3.1 , OZLCI2019, Reference[7]
	Light fuel oil	MJ	0.456	Ecoinvent 3.1 , OZLCI2019, Reference[7]
	Natural gas	MJ	1.27	Ecoinvent 3.1 , OZLCI2019, Reference[7]
Output	Sodium Silicate	Kg	1.00	Ecoinvent 3.1 , OZLCI2019, Reference[7]
	Inorganic salts and acids	g	1.034	Ecoinvent 3.1 , OZLCI2019, Reference[7]
	Metals	g	0.0173	Ecoinvent 3.1 , OZLCI2019, Reference[7]
	Chlorides	g	6.316	Ecoinvent 3.1 , OZLCI2019, Reference[7]
	Suspended solids	g	0.892	Ecoinvent 3.1 , OZLCI2019, Reference[7]
	Dust	g	0.667	Ecoinvent 3.1 , OZLCI2019, Reference[7]
	Non-methane volatile organic component (NMVOC)	g	2.451	Ecoinvent 3.1 , OZLCI2019, Reference[7]
	Mineral waste (ash)	g	0.0202	Ecoinvent 3.1 , OZLCI2019, Reference[7]
Emission	Carbon dioxide, fossil	kg	0.288	Ecoinvent 3.1 , OZLCI2019, Reference[7]
	Nitrogen oxides , NO	g	1.748	Ecoinvent 3.1 , OZLCI2019, Reference[7]
	Sulfur oxides	g	2.186	Ecoinvent 3.1 , OZLCI2019, Reference[7]

	Carbon monoxide	g	0.218	Ecoinvent 3.1 , OZLCI2019, Reference[7]

Table 7: Input and output inventories for 1kg sodium aluminate production

	**Category**	**Unit**	**Value**	**Database source for Provider**
Input	Bauxite	Kg	1.9	Ecoinvent 3.1, OZLCI2019, Reference[8]
	Steam	Kg	4.48	Ecoinvent 3.1 , OZLCI2019
	Sodium hydroxide	Kg	1.6	OZLCI2019
	Demineralized water	m3	0.4	OZLCI2019
	Electricity	MJ	7.44	Electricity grid mix, AC, < 1kV
Output	Sodium Aluminate	Kg	1.00	Reference [9]
	Red mud (dry)	kg	1.8	OZLCI2019, Reference [9]
Emission	Carbon dioxide, fossil	kg	0.8	Reference [9]

Table S8: Isotherms at 3 different temperatures(25 °C - 60 °C) and the dual site Langmuir fitting values

https://docs.google.com/spreadsheets/d/1GybRSNRkiNCSuc45NeOd8owEQzvJ5CVM/edit?usp=drive_link&ouid=100721013064026322254&rtpof=true&sd=true

Table S9: Unit prices and material cost contributions for the chemical based synthesis of 1 kg Zeolite 13X.

**Item**	**Amount**	**Unit price**	**Cost (EUR)**
Sodium silicate	1.517 kg	0.60 €/kg	0.91

Sodium aluminate	0.540 kg	1.20 €/kg	0.65
NaOH	0.443 kg	0.50 €/kg	0.22
Demineralized water	0.00878 m³	1.0 €/m³	0.009
Electricity	122.8 MJ = 34.1 kWh	0.12 €/kWh	4.09

Table S10: Unit prices and material cost contributions for the clay based synthesis of 1 kg Zeolite 13X

Input	Amount	Unit price	Cost (€)
Bentonite clay	0.417 kg	0.10 €/kg	0.042
NaOH	0.583 kg	0.50 €/kg	0.292
Demineralized water	0.0096 m³	1.0 €/m³	0.01
Electricity	108 MJ = 30.0 kWh	0.12 €/kWh	3.6

Figure S4: Expanded view of the low relative pressure region ($p/p_0 \leq 0.10$) of the nitrogen adsorption-desorption isotherms shown in Figure 5a.

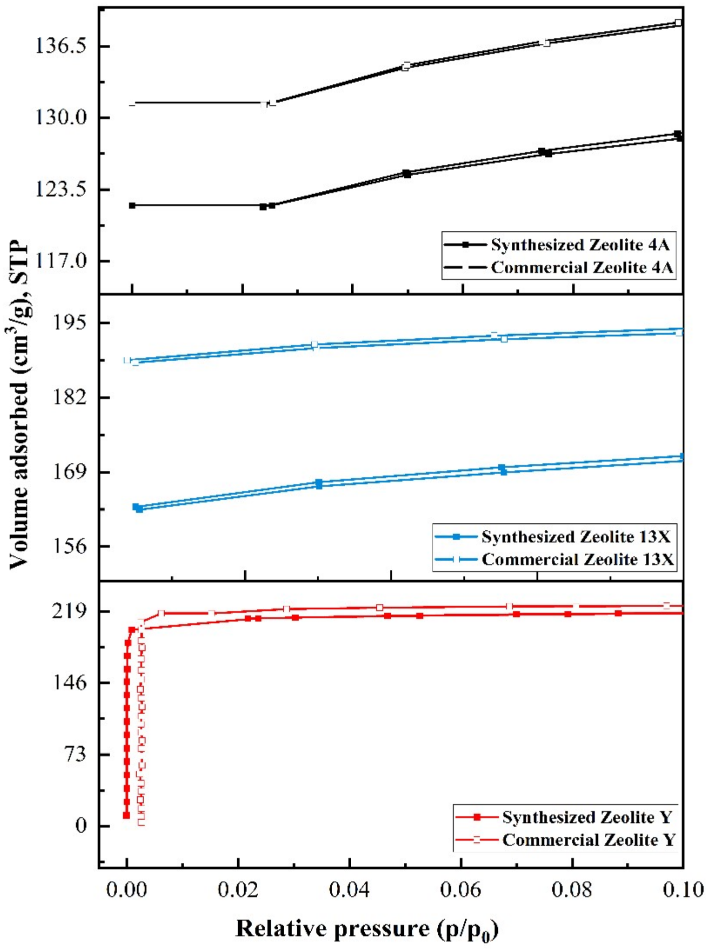

Reference

(1) Zukal, A.; Arean, C. O.; Delgado, M. R.; Nachtigall, P.; Pulido, A.; Mayerová, J.; Čejka, J. Combined Volumetric, Infrared Spectroscopic and Theoretical Investigation of CO2 Adsorption on Na-A Zeolite. *Microporous and Mesoporous Materials* **2011**, *146* (1), 97–105. https://doi.org/10.1016/j.micromeso.2011.03.034.

(2) Hyla, A. S.; Fang, H.; Boulfelfel, S. E.; Muraro, G.; Paur, C.; Strohmaier, K.; Ravikovitch, P. I.; Sholl, D. S. Significant Temperature Dependence of the Isosteric Heats of Adsorption of Gases in Zeolites Demonstrated by Experiments and Molecular Simulations. *J. Phys. Chem. C* **2019**, *123* (33), 20405–20412. https://doi.org/10.1021/acs.jpcc.9b05758.

(3)Golipour, H.; Mokhtarani, B.; Mafi, M.; Khadivi, M.; Godini, H. R. Systematic Measurements of CH4 and CO2 Adsorption Isotherms on Cation-Exchanged Zeolites 13X. *J. Chem. Eng. Data* **2019**, *64* (10), 4412–4423. https://doi.org/10.1021/acs.jced.9b00473.

(4) Cavenati, S.; Grande, A.; Rodrigues, A. E. Adsorption Equilibrium of Methane, Carbon Dioxide, and Nitrogen on Zeolite 13X at High Pressures. *Journal of Chemical & Engineering Data* **2004**, *49*, 1095–1101.

(5) Shao, W.; Zhang, L.; Li, L.; Lee, R. L. Adsorption of CO2 and N2 on Synthesized NaY Zeolite at High Temperatures. *Adsorption* **2009**, *15* (5), 497–505. https://doi.org/10.1007/s10450-009-9200-y.

(6) Hemant Mishra; Sanju Kumari; Pramod Kumar; Prashant Mishra. 13x Zeolite, Synthesis Method and Use Thereof. WO2023119309A1. https://patents.google.com/patent/WO2023119309A1/en.

(7) Fawer, M.; Concannon, M.; Rieber, W. Life Cycle Inventories for the Production of Sodium Silicates. *The International Journal of Life Cycle Assessment* **1999**, *4*, 207–212.

(8) Farjana, S. H.; Huda, N.; Mahmud, M. A. P. Impacts of Aluminum Production: A Cradle to Gate Investigation Using Life-Cycle Assessment. *Science of The Total Environment* **2019**, *663*, 958–970. https://doi.org/10.1016/j.scitotenv.2019.01.400.

(9) Homa, J.; Hoffmann, E. *Life Cycle Analysis of Leading Coagulants: Executive Summary*; INCOPA: Karlsruhe, 2014.
